# Racial and Ethnic Inequities to Cumulative Environmental and Occupational Impacts in Michigan

**DOI:** 10.1029/2025GH001482

**Published:** 2025-06-20

**Authors:** Abas Shkembi, Sung Kyun Park, Jon Zelner, Richard Neitzel

**Affiliations:** ^1^ Department of Environmental Health Sciences University of Michigan School of Public Health Ann Arbor MI USA; ^2^ Department of Epidemiology University of Michigan School of Public Health Ann Arbor MI USA; ^3^ Center for Social Epidemiology and Population Health University of Michigan School of Public Health Ann Arbor MI USA

**Keywords:** cumulative impacts, environmental justice, occupational justice, neighborhood, redlining

## Abstract

The contribution of occupational exposures to the extent of cumulative environmental impacts, and their implications for environmental justice (EJ), have not been investigated. We (a) characterized communities with cumulatively high occupational and environmental exposures, (b) examined whether marginalized, historically redlined neighborhoods were disproportionately affected by these exposures, and (c) evaluated the implications of failing to consider workplace exposures in EJ screening tools in Michigan. At the census tract‐level, we combined occupational exposure estimates of six common workplace hazards, environmental exposures from EJScreen and the National Transportation Noise Map, demographic information from the American Community Survey, and redlining information from the 1930s Home Owners' Loan Corporation maps to test the first two objectives using supervised and unsupervised statistical methods. The last objective incorporated the occupational indicators into the Michigan‐specific EJ screening tool (MiEJScreen) to test the third objective. Among 2,772 Michigan census tracts, 738 (27%) had cumulatively high occupational and environmental exposures, primarily in urban areas. Tracts with >90% (compared to <10%) of racial and ethnic minority individuals had 2.31 (95% CI: 1.78–3.03) times higher odds of cumulatively high exposures. A simultaneous increase to the 90th percentile (relative to the 50th) in all 13 occupational and environmental exposures was associated with 2.47 (95% CI: 1.20–5.36) times higher odds of a tract having been historically redlined. Not incorporating occupational exposures into the MiEJScreen would overlook 90 census tracts with cumulatively high environmental and occupational impacts, affecting around 255,000 individuals. Ignoring occupational exposures in cumulative environmental impact assessments may overlook important EJ hotspots.

## Key Points

- Previous research has understudied the joint contributions of occupational and environmental exposures together
- Racial and ethnic minority communities in Michigan are burdened by cumulatively high occupational and environmental exposures
- Not incorporating occupational exposures may be making current efforts to address environmental injustices ineffective

## 1 Introduction

For decades, the field of environmental health sciences has recognized the importance of and sought to characterize cumulative impacts of multiple, overlapping chemical and non‐chemical hazards (Solomon et al., 2016). With increasing capabilities to sample for complex mixtures of chemicals and pollutants, handle big quantities of data, and analyze the health effects of these complex mixtures (Bobb et al., 2015; Keil et al., 2020; Park et al., 2017), the field is advancing toward this goal. Despite these advancements, investigating cumulative impacts from multiple sources of environmental exposure—community, occupational, indoor, and personal consumer products—on human health remains fragmented. In particular, occupational and community (henceforth used interchangeably with “environmental,” although recognizing that environmental exposures encompass occupational exposures) exposures are not typically examined cumulatively by researchers or practitioners, nor are policies enacted by the US Environmental Protection Agency (EPA) or Occupational Safety and Health Administration (OSHA) to protect against potential cumulative exposures. This is likely due to a myriad of social and political pressures, like the separation of responsibilities between the EPA and OSHA. Even the separation of funding between the US National Institute of Environmental Health Sciences (NIEHS) and National Institute for Occupational Safety and Health (NIOSH) makes it difficult for the NIEHS to fund research with a major occupational focus, and vice versa for NIOSH. Ignoring occupational exposures when assessing cumulative environmental impacts may underestimate the total burden on a community. As a consequence, we risk getting biased estimates of the impact of spatially localized environmental exposures on adverse health outcomes.

**Figure 1:**
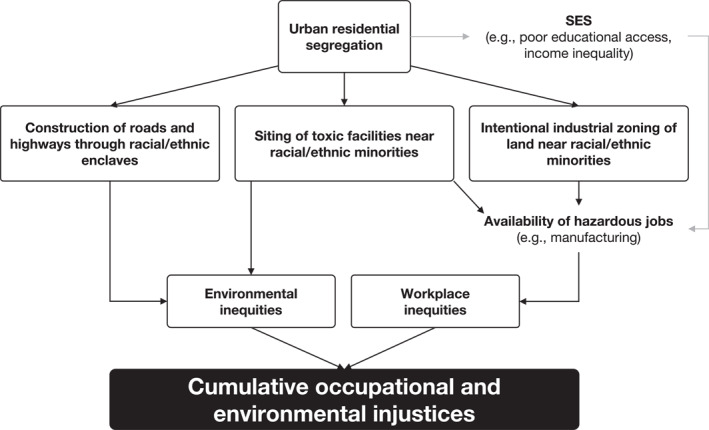
Simplified, directed, acyclic conceptual framework of the creation of cumulative occupational and environmental injustices in urban contexts. Note that many of the relationships in the framework are bi‐directional in reality (e.g., residential segregation influenced the construction of highways through racial and ethnic enclaves, which in turn deepened residential segregation).

Examining environmental and occupational exposures separately may bias these assessments for two main reasons. First, people may be exposed to the same hazards in the workplace at levels orders of magnitude higher than in their communities. For example, the EPA's 24‐hr limit to PM_10_ is 150 μg/m^3^ (Environmental Protection Agency, 2013), yet workers in the US can still be exposed daily to respirable particulates (those <10 μm in diameter) at levels as high as 5,000 μg/m^3^ in 8 hr (Occupational Safety and Health Administration, 1999). Second, individuals most burdened in their community are likely to be among those experiencing the greatest intensity of occupational exposures. For example, one case study in the Bronx, NY found that neighborhoods with a high proportion of workers employed in dangerous occupations, such as manufacturing, also had elevated exposure in their residential context (Maroko & Pavilonis, 2018), suggesting cumulative exposures. More robust evidence in the literature supporting this notion is limited, although Figure 1 illustrates our hypothesized conceptual framework on why this phenomenon may be true elsewhere in the US.

While residential segregation, both historical and current, has long been linked to environmental inequities (the left side of Figure 1) (R. D. Bullard, 1983; Morello‐Frosch & Jesdale, 2006; Swope et al., 2022), residential segregation has also been linked to occupational segregation. The COVID‐19 pandemic demonstrated this, where Black, immigrant, and Indigenous communities were disproportionately burdened by risk of transmission partly because they are more likely to work in “essential”, manual labor, lower wage jobs (McClure et al., 2020). These disparities were even documented in early pioneering environmental justice (EJ) research studies (R. D. Bullard & Wright, 1993; Davis & Rowland, 1980; Friedman‐Jiménez, 1994; Wright & Bullard, 1990). Given various racist land use decisions stemming from residential segregation have resulted in environmental inequities, such as the siting of toxic facilities and intentional industrial zoning (R. Bullard, 1993; Cushing et al., 2022; Gonzalez et al., 2022; Karas, 2015), these same decisions may have impacted the availability of hazardous jobs and worsened socioeconomic conditions, resulting in workplace inequities as well. Thus, the potential for a cumulative environmental and occupational injustices may exist, although the extent and nature of these injustices has not been previously characterized. There is a need to account for people's exposures to various chemical and non‐chemical hazards both in their home community as well as when people are at work to truly understand place‐based burdens. Such assessments have not been possible previously because our understanding of the exposures that residents experience away from home while they are at work has been limited, unlike our understanding of environmental exposures; however, our team recently developed census tract‐level estimates of six common occupational hazards across the US (Shkembi, Zelner, et al., 2024).

Building off of our conceptual framework and these recently developed estimates, this study investigates cumulative environmental and occupational impacts in Michigan and associated racial and ethnic inequities. We situate this investigation in the state of Michigan due to its unique history of intense industrialization throughout the twentieth century, racialized residential segregation, ongoing environmental pollution, and government mismanagement of public works throughout cities in Michigan that have become hallmarks of environmental injustices, especially in Detroit and Flint (Benz, 2019; Downey, 2006; Schulz et al., 2016; Smith, 2007). We examine whether there are census tracts in Michigan with both high environmental burdens and high occupational burdens. We hypothesize that these census tracts are located in urban areas of Michigan. We then examine whether communities with a high proportion of marginalized groups (i.e., racial and ethnic minority, low‐income, and low educational attainment) are disproportionately impacted by cumulatively high environmental and occupational exposures, and whether urban residential segregation (assessed through historical redlining) may be associated with this cumulative injustice.

Lastly, we provide a case study of the implications of failing to consider workplace exposures in EJ screening tools. Since occupational inequities are an essential component of environmental justice, we sought to take a first step toward demonstrating the need to develop cumulative impacts policy and refine decision‐making tools that account for exposures to not only environmental hazards, but also hazards at work. The advantage of cumulative impact tools such as the EPA's EJScreen and CalEnviroScreen is that they evaluate environmental justice across the cumulative impacts of many environmental hazards and facilitate the addition of other relevant hazards for a particular state (Lee, 2020). Our current study made use of this fact by incorporating the six spatial metrics of occupational hazards for each census tract in Michigan into the MiEJScreen (an EJ screening tool for neighborhoods in Michigan) (Office of the Environmental Justice Public Advocate, 2022). We hypothesize that not incorporating occupational exposures in this EJ screening tool may misidentify the most burdened communities in Michigan.

## 2 Methods

This ecological study of occupational and environmental justice in Michigan was conducted between 2021 and 2024. We utilized six occupational hazard indicators at the census tract level that were previously created by our team (Shkembi, Zelner, et al., 2024), which were combined with pre‐existing census tract‐level environmental indicators made available by the EPA EJScreen (United States Environmental Protection Agency, 2020). Disparities by race/ethnicity, income, and educational attainment were investigated through census tract‐level estimates from the EPA EJScreen that were derived from the US American Community Survey (ACS). Differences in occupational and environmental indicators as a function of historical redlining were examined using 1930s Home Owners' Loan Corporation (HOLC) grades at the census tract‐level (Meier & Mitchell, 2021). Our analyses concluded with a comparison of how state‐specific EJ screening tools (in this case, the MiEJScreen) that seek to identify EJ “hotspots” may change if occupational indicators were considered alongside environmental indicators. All of the data used in this study were de‐identified, secondary data, and no human subjects participated in any aspect of this particular study.

### 2.1 Occupational Exposures

In 2021, our team previously developed six occupational hazard indicators for each census tract across the US. Information on how each indicator was constructed was previously detailed (Shkembi, Zelner, et al., 2024). Additional details have been provided in Appendix A. The occupational hazard indicators were developed by leveraging three existing data resources: (a) employment counts from the 5‐year, 2015–2019 US Census ACS (www.census.gov/programs‐surveys/acs), (b) self‐reported occupational exposure data from the Department of Labor (DoL) Occupational Information Network (O*NET; www.onetcenter.org/), and (c) a US/Canada noise job exposure matrix (NoiseJEM; www.noisejem.sph.umich.edu/). The six occupational indicators across Michigan census tracts used in this study include five indicating the average frequency of days exposed to (a) contaminants, (b) hazardous equipment, (c) hazardous conditions, (d) disease/infections, and (e) close physical proximity to other workers, and one indicating the (f) prevalence of hazardous workplace noise exposure. These indicators are not an estimate of work‐related exposures occurring among workplaces/facilities in a given census tract. Rather, they are an estimate of people's personal exposure to hazards at their workplace, which may or may not be in the same census tract where an individual lives.

### 2.2 Environmental and Demographic Indicators

The nationwide EPA EJScreen v1.0 provides environmental and demographic indicators at the census tract level from 2013 to 2019 (United States Environmental Protection Agency, 2019, 2020). This includes six demographic indicators which come from the US ACS: % low‐income, % racial and ethnic minority (defined as individuals who list their racial status as a race other than White‐alone and/or list their ethnicity as Hispanic or Latino), % with less than a high school education, % linguistically isolated, % <5 years of age, and % >64 years of age. We did not explore further breakdowns in racial and ethnic groups because our study was motivated by environmental racism broadly, which disproportionately burdens all racial and ethnic minority groups.

There are 11 environmental indicators, split into environmental exposures (i.e., indicators that represent exposure to pollutants) and environmental effects (i.e., indicators that represent *potential* exposure to pollutants, such as the proximity to a hazardous waste facility). We only included the six environmental exposure indicators in this study for two reasons. First, the occupational indicators also represent exposure to hazards, not potential exposure, meaning conceptually the environmental exposures are more similar to the occupational indicators than the environmental effects. Second, we sought a balance between the number of occupational environmental indicators as some of the findings from our statistical approaches (e.g., the *k*‐means clustering, the boosted regression tree) would be biased simply by the number of variables included; if we assessed 11 environmental indicators alongside only six occupational indicators, our results could be skewed more toward environmental trends than occupational trends. The six environmental exposure indicators are air toxics cancer risk; respiratory hazard index; diesel PM; PM_2.5_; ozone; and traffic volume. Each of the environmental and demographic indicators are provided in raw measurement averages for each census tract.

In addition to these metrics, we constructed an additional environmental indicator to account for the impact of community noise exposure. Noise pollution was included in this study due to the widespread, but poorly appreciated, environmental harm it creates in terms of both hearing‐ and non‐hearing‐related health outcomes (e.g., cardiovascular disease, diabetes), as well as indications of noise‐related environmental injustices (Casey et al., 2017; Collins & Grineski, 2024; Rowangould, 2013). We amassed 24‐hr traffic‐related noise exposure point‐estimates from the Department of Transportation (DOT) National Transportation Noise Map (NTNM) (U.S. Department of Transportation, 2020). These reflect road, air, and rail traffic noise and range from 45 dBA to >100 dBA. We used a Monte Carlo simulation approach to aggregate the noise data to the census tract‐level for integration with the other environmental and occupational indicators. We randomly sampled transportation noise measurements with replacement from the DOT NTNM equal to the number of residents in each census tract to create a distribution of environmental exposure for each census tract. We repeated this sampling procedure 100 times to generate 100 exposure distributions for each census tract. We deemed exposures >70 dBA over a 24‐hr period as hazardous (World Health Organization, 2018). Using the 100 Monte Carlo simulations, we calculated the mean percent of residents potentially exposed to hazardous transportation noise and used this mean in subsequent analyses.

### 2.3 Historical Redlining

Following our conceptual framework in Figure 1, we sought to test whether urban residential segregation is an important upstream factor than could lead to cumulative occupational and environmental inequities. Many institutional (e.g., racial covenants, racialized law enforcement) and informal (e.g., mob violence) racist practices led to urban residential segregation. One racist practice known as historical redlining, which involved the systematic denial of home mortgages to racial and ethnic minorities, has been consistently associated with poorer environmental conditions today (Swope et al., 2022). A commonly used measure of historical redlining is 1930s and 1940s HOLC maps. These maps serve as a reflection of systemic, residential segregation at the time among a subset of neighborhoods in the US. Using a marker of past residential segregation, rather than current‐day segregation (e.g., dissimilarity index), ensures our analyses match temporally with our hypothesized conceptual framework.

Since neighborhoods defined in these historical maps do not overlap with current‐day census tracts, we determined which neighborhoods were historically redlined using historical redlining scores (Meier & Mitchell, 2021). These provide an area‐weighted score (from 1 to 4) to each census tract based on the proportion of HOLC grades which overlap current‐day census tracts, reflecting the original HOLC grades from A to D. An HOLC grade of “D” represents historically redlined neighborhoods in this study. Specifically for Michigan, HOLC maps were created for only 11 cities (Detroit, Pontiac, Flint, Saginaw, Bay City, Jackson, Lansing, Battle Creek, Kalamazoo, Grand Rapids, and Muskegon), reflecting 729 (or 26%) of all Michigan census tracts.

### 2.4 MiEJScreen

We investigated whether including occupational indicators into cumulative environmental justice screening tools would significantly alter which communities are identified as highly burdened. Since the nationwide EJScreen does not calculate an overall EJ score for each census tract, we used the drafted Michigan Environmental Justice Screening tool in 2023 (MiEJScreen) (Office of the Environmental Justice Public Advocate, 2022), which does calculate an overall EJ score (from 0 to 100, where 100 reflects the most burdened census tracts). As of 2025, the MiEJScreen is no longer a draft and an official version has been publicly released. The method to calculate an overall EJ score for each census tract is based on California's EJ screening tool (CalEnvironScreen) (August et al., 2021). The score is a multiplication of the (a) Environmental Conditions subscore (the average environmental exposures and effects), and (b) Population Characteristics subscore (the average of sensitive populations and socioeconomic factors).

**Figure 2:**
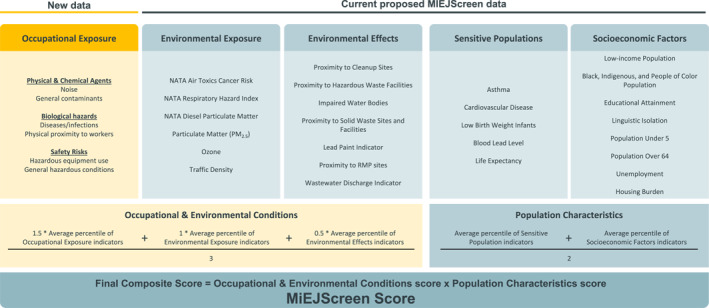
Construction of the final composite MiEJScreen score incorporating the six additional occupational exposure indicators, utilizing the same methodology used in the original MiEJScreen score (which currently does not include occupational indicators). Note that a weighting factor of 1.5 was assigned to the average percentile of occupational exposure indicators within the Occupational & Environmental Conditions score, so that the environmental conditions were equally weighted to the occupational conditions.

We recalculated the overall score using the exact same methodology laid out MiEJScreen Draft Technical Report (Office of the Environmental Justice Public Advocate, 2022) and the same data available in the MiEJScreen by incorporating the six occupational indicators to each MI census tract (details are depicted in Figure 2). For the purpose of considering occupational exposures cumulatively alongside environmental conditions, our recalculated score altered the “Environmental Conditions” subscore to an “Occupational and Environmental Conditions” subscore. The original “Environmental Conditions” subscore weighted the “Environmental Effects” indicators at 0.5 of the “Environmental Exposure” indicators. As a result, our recalculated score incorporated a 1.5 weight for the average percentile of the six occupational indicators. Our rationale is that the “Occupational Exposure” indicators should be weighted half of the overall “Occupational and Environmental Conditions” subscore since the summation of the “Environmental Exposures” and “Environmental Effects” also equates to a 1.5 weight of the subscore. The “Population Characteristics” subscore was not altered.

### 2.5 Statistical Analysis

All analyses were conducted in R v4.0.2. Maps were constructed using the “tigris” R package (Walker, 2022). Summary statistics (medians, interquartile ranges [IQRs], percentiles, Pearson's correlation coefficients) were used to describe the occupational and environmental indicator data. To examine crude trends by race and ethnicity with each of the occupational and environmental indicators, inequality curves were developed.

The first statistical analysis examined whether racial and ethnic minority individuals are disproportionately impacted by workplace exposures. Environmental injustices are well‐documented, while occupational injustices are not; thus, we felt it important to investigate occupational exposure inequities independent of environmental exposures. Six, separate, Poisson conditional autoregressive (CAR) models were constructed for each of the six occupational indicators. CAR models were used because a contiguity matrix can be specified for areal data (like the census tract‐level indicators in this analysis) that accounts for spatial autocorrelation between neighboring census tracts (Besag, 1974; Schmidt & Nobre, 2018). Specifically for this analysis, we used a queen‐based contiguity matrix, meaning any two census tracts that share a common border in any direction would be considered neighbors. The CAR model was preferred to more commonly used simultaneous autoregressive (SAR) models when analyzing spatial data, such as the spatial error or spatial lag model, because the occupational indicators represent the typical work‐related exposures for people living in a census tract. As such, we consider the spatial dependency of the occupational indicators to be more of a function of space itself (first order spatial dependency, appropriate for CAR models), than a function of neighboring observations (second order spatial dependency, appropriate for SAR models). Poisson regression was chosen because five of the frequency indicators were counts; similarly, the occupational noise prevalence indicator was modeled on the count of workers exposed but with an offset to account for differences in the total number of workers of a census tract. Each of these indicators served as separate outcomes and the census tract‐level percentage of racial and ethnic minority individuals as the primary independent variable. Each model adjusted for the census tract‐level population density, percent under 5 years old, and percent above 64 years old.

The second statistical analysis examined whether there are any census tracts clusters with both high workplace and environmental exposures. A *k*‐means clustering algorithm was constructed using the six occupational indicators and the seven environmental exposure indicators to characterize the typical occupational and environmental exposures experienced by residents of tracts in Michigan. The *k*‐means clustering algorithm identifies *k* groups with similar occupational and environmental exposures, where the number of groups is unknown ahead of time and can be estimated simultaneously with cluster memberships. The 13 indicators were normalized (mean‐centered and variance standardized, to reduce bias from unequal variance) prior to running the algorithm, and the optimal number of clusters was selected based on the average silhouette method (Rousseeuw, 1987). We did not consider any dimension reduction of highly correlated indicators because potential collinearity is a feature of this analysis of multiple, overlapping exposures that lead to cumulative inequities.

The third primary statistical analysis examined the relationship between the proportion of marginalized residents in a census tract (i.e., any of racial and ethnic minority individuals, low‐income individuals, and individuals without high school diplomas) and the odds of being in a cluster with high workplace and environmental exposures. We used a gradient boosted regression tree model to evaluate (a) whether tracts with simultaneous, quantile increases in the percentage of racial and ethnic minority individuals, low‐income individuals, and individuals without high school diplomas was associated with higher odds of being in a cluster with high workplace and environmental exposures compared to all other census tracts, and (b) the univariate relationships of these three demographic indicators. A gradient boosted regression tree model was chosen over traditional logistic regression because we assumed that the effect of race, ethnicity, income, and educational attainment on exposures are non‐additive and non‐linear, while traditional regression techniques assume independence and linearity. Other models capable of handling non‐additive and non‐linear effects, such as Bayesian Kernel Machine Regression, were not preferred because of their computational inefficiency in handling large sample sizes. The model adjusted for the census tract‐level population density, percent under 5 years old, percent above 64 years old, the county, and the centroid (in latitude/longitude). Odds ratios and 95% confidence intervals (CIs) were estimated using 100 bootstraps of a boosted regression tree model grown 500 times.

The fourth primary statistical analysis examined whether historically redlined census tracts in Michigan continue to be exposed to cumulatively high occupational and environmental exposures. Among the subset of census tracts in Michigan that overlap with the 1930s HOLC maps (*n* = 729 of 2,772 total tracts), we constructed another gradient boosted regression tree model with historically redlined tracts (Grade D) as the outcome and the six occupational and seven environmental indicators as predictors. Using 1,000 bootstrap replicates, we investigated whether simultaneous, quantile increases in the 13 occupational and environmental indicators were associated with increased odds of a tract having been historically redlined (Grade D) compared to non‐historically redlined neighborhoods (Grades A‐C). Swapping the dependent and independent variables in this manner (i.e., we would normally model historical redlining as the main independent variable and the occupational/environmental indicators as the main dependent variables) allows us to pool all of the occupational and environmental exposures into one model to examine whether historically redlined tracts are cumulatively exposed to occupational and environmental exposures today (Shkembi, Smith, & Neitzel, 2024). The use of a gradient boosted regression tree model was similarly motivated by the assumptions of non‐additivity and non‐linearity in the environmental and occupational indicators, as well as its computational efficiency. The ability to handle non‐additivity is a crucial advantage of this model for assessing cumulative impacts because many of the occupational and environmental indicators are inherently dependent, overlapping and colinear with each other. Another advantage of this model was the ability to identify the most pervasive environmental and occupational exposures driving cumulative inequities between redlined and non‐redlined neighborhoods in Michigan. The model adjusted for the census tract‐level percentage of low‐income individuals and those without a high school diploma to examine whether these cumulative injustices persist independent of differences in the socioeconomic status of a census tract.

Finally, we examined how the MiEJScreen score for each tract would change if the six occupational indicators were included. Scores ≥90 are considered the most burdened environmental justice communities in the MiEJScreen. We identified four types of census tracts: (a) those with scores ≥90 both in the original MiEJScreen as well as after inclusion the occupational indicators, (b) those with scores ≥90 in the original MiEJScreen but <90 after including the occupational indicators, (c) those with scores <90 in the original MiEJScreen but ≥90 after including the occupational indicators, and (d) those with scores <90 in both scenarios. The third type of census tracts reflects those that wouldn't be identified as the most burdened if occupational exposures are not considered and represent the main interest in this analysis.

### 2.6 Sensitivity Analysis

The overall MiEJScreen score for a given census tract is sensitive to the choice of weights. While we believe inclusion of the occupational indicators alongside the environmental exposures and effects to construct an “Occupational and Environmental Conditions” subscore is ideal to assess cumulative impacts of exposures, it may also be reasonable to incorporate the occupational indicators in the “Population Characteristics” subscore since these indicators are an estimate of a subpopulation's personal exposure to hazards at work outside their home tracts. Thus, we replicated the final analysis of the overall MiEJScreen score by altering the “Population Characteristics” subscore as the mean of the average percentiles of “Sensitive Populations”, “Occupational Exposures”, and “Socioeconomic Factors” indicators, each equally weighted (see Figure S1 in Supporting Information S1).

## 3 Results

### 3.1 Overview of Study Data

In this study, we evaluated six novel metrics of occupational exposures alongside seven environmental exposures from the EJScreen among 2,772 Michigan census tracts with at least 20 working residents. In a standard 250‐day work‐year, workers in the median Michigan census tract had spent 88 (IQR: 82–93) days at work physically close to other workers, 58 (IQR: 48–67) days exposed to chemical contaminants, 33 (IQR: 26–39) days using hazardous equipment, 23 (IQR: 19–26) days working in hazardous conditions, and 22 (IQR: 19–26) days exposed to disease/infections (Table 1). The median census tract had 12.9% (IQR: 9.1%–16.3%) of its employed residents exposed to hazardous levels of noise over a typical work year. Figure S2 in Supporting Information S1 displays that higher occupational exposures are generally found in both urban and rural areas of Michigan. Table 1 summarizes the remaining environmental exposures and sociodemographic indicators. Across all of Michigan, occupational exposures indicated weak/no correlation with environmental indicators (−0.25 < *r* < 0.03; Figure S3i in Supporting Information S1). The average percentile of the occupational exposures and the environmental exposures displayed a C‐shaped relationship (Figure S3ii in Supporting Information S1), which may explain why weak associations were observed. This C‐shaped relationship was driven by differences in urban versus rural exposures.

**Table 1:** Summary Statistics on the Sociodemographic, Occupational Exposure, and Environmental Exposure Indicators Included in This Study, Across 2,772 Census Tracts in Michigan With ≥20 Working Residents

Indicator	Data sources	Median (IQR)	Range
Sociodemographic			
Racial and ethnic minority (% of population)	EJScreen^a^	15.0% (7.0–38.5)	0%–100%
Low‐income (% of population)	EJScreen^a^	31.1% (19.5–46.1)	0%–100%
No HS diploma (% of population)	EJScreen^a^	6.0% (3.6–9.4)	0%–43.7%
Occupational^b^			
Hazardous noise (% of working population)	ACS + NoiseJEM + O*NET	12.9% (9.1%–16.3%)	0.3%–35.8%
Physical proximity (in days)^c^	ACS + O*NET	88 (82–93)	28–166
Chemical contaminant exposure (in days)	ACS + O*NET	58 (48–67)	15–137
Hazardous equipment use (in days)	ACS + O*NET	33 (26–39)	7–84
Hazardous conditions (in days)	ACS + O*NET	23 (19–26)	7–52
Disease/infection exposure (in days)	ACS + O*NET	22 (19–26)	4–57
Environmental			
PM_2.5_ (μg/m^3^)	EJScreen	8.2 (7.6–9.2)	5.0–9.6
Diesel PM (μg/m^3^)	EJScreen	0.3 (0.2–0.5)	0.04–0.9
Ozone (ppb)	EJScreen	43.4 (42.4–44.4)	32.4–48.3
Air toxics cancer risk (persons per million lifetime)	EJScreen	23.8 (20.6–27.9)	13.2–118
Respiratory hazard index (unitless)	EJScreen	0.3 (0.2–0.4)	0.1–0.5
Traffic volume (count within 500 m)	EJScreen	356 (59–974)	0–13,257
Transportation noise indicator (% of population exposed)	DOT National Transportation Noise Map	0.1% (0–5.5)	0%–31.3%

*Note.* HS, high school; EJScreen, Environmental Justice Screening Tool; ACS, American Community Survey; NoiseJEM, Noise job exposure matrix; O*NET, Occupational Information Network; PM, particulate matter; DOT, Department of Transportation.

^a^ Originally calculated using US American Community Survey data.

^b^ The occupational indicators should be interpreted as exposure among the population of workers living in a given census tract.

^c^ The “physical proximity” measure is an indicator of the days spent in close physical proximity to other workers.

### 3.2 Association Between Race, Ethnicity and Occupational Exposures

**Figure 3:**
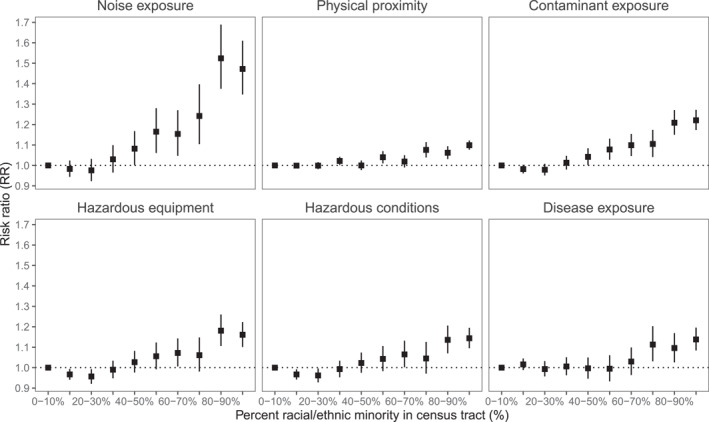
Risk ratio and 95% confidence intervals from conditional autoregressive Poisson regression models examining census tract‐level race and ethnicity and occupational exposures. Reference level set to 0%–10% racial and ethnic minority individuals in a census tract. Models adjusted for population density, percent under 5 years old, and percent above 64 years old. Racial and ethnic minority individuals defined as any individual who is not non‐Hispanic White. See Table S1 in Supporting Information S1 for details on the effect estimates.

Inequality curves demonstrated that both occupational and environmental exposures have substantial inequality toward racial and ethnic minority individuals (Figure S4 in Supporting Information S1), with the inequality more pronounced among the environmental exposures than the occupational exposures. Particular for occupational exposures, census tracts with a higher proportion of marginalized groups by race and ethnicity, income, and educational attainment were generally associated with higher occupational exposures, except to disease/infection (Figure S5 in Supporting Information S1). Specifically by race and ethnicity, using conditional autoregressive Poisson regression models adjusted for population density, percent under 5 years old, and percent above 64 years old, we observed that census tracts with the highest proportion of racial and ethnic minority individuals had the highest risk of exposure to all occupational indicators (Figure 3 and Table S1 in Supporting Information S1). Census tracts with 90%–100% of racial and ethnic minority individuals had significantly higher risk of occupational noise exposure (RR = 1.47; 95% CI: 1.35–1.61), chemical contaminant exposure (RR = 1.22; 95% CI: 1.17–1.27), use of hazardous workplace equipment (RR = 1.16; 95% CI: 1.10–1.22), disease/infection exposure (RR = 1.14; 95% CI: 1.08–1.20), working in hazardous conditions (RR = 1.14; 95% CI: 1.09–1.19), and physical proximity to others at work (RR = 1.10; 95% CI: 1.03–1.09).

### 3.3 Cumulative Occupational and Environmental Exposures

**Figure 4:**
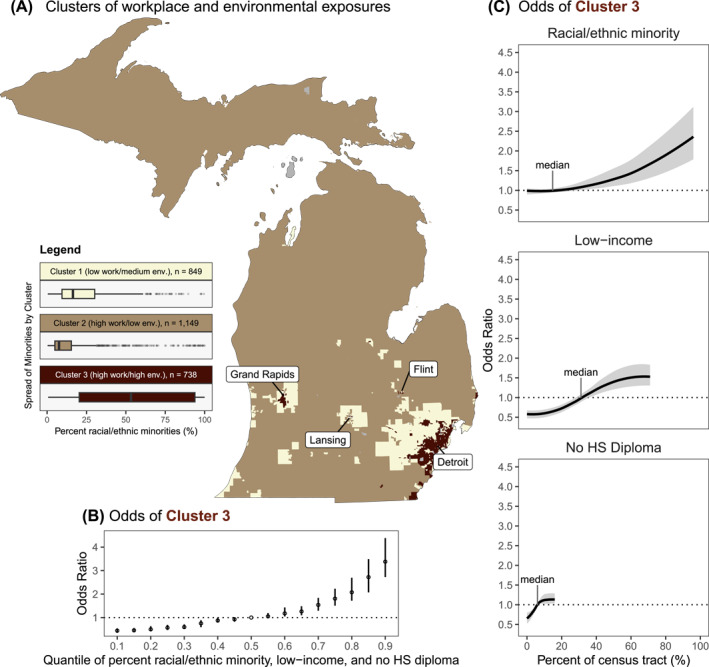
Census tracts in Michigan with cumulatively high workplace and environmental exposures and their relationship with marginalized communities. (a) Census tract clusters of six occupational exposures (average frequency of days exposed to contaminants, hazardous equipment, hazardous conditions, disease/infections, and physical proximity with other workers, and the prevalence of hazardous workplace noise exposure) and seven environmental exposures (air toxics cancer risk; respiratory hazard index; diesel PM; PM_2.5_; ozone; traffic volume; and a transportation noise indicator) identified using a *k*‐means clustering algorithm: Cluster 1—tracts with low workplace and medium environmental exposures, colored in yellow; Cluster 2—tracts with high workplace exposures and low environmental exposures, colored in beige; Cluster 3—tracts with high workplace exposures and high environmental exposures, colored in maroon. (b) Odds (95% confidence interval) of a Cluster 3 census tract (compared to Clusters 1 and 2) with increasing percentage of racial and ethnic minorities, low‐income individuals, and individuals without a high school diploma. Effect reflects the ratio in odds when the three sociodemographic indicators are fixed at a specific quantile (ranging from 0.1 to 0.9), as compared to when the factors are fixed at their median value. (c) Univariate relationships between the three sociodemographic indicators and the odds (95% confidence interval shaded in gray) of a census tract in Cluster 3. The dotted lines represent the null effect. Odds ratios in (b) and (c) were modeled using a gradient boosted regression tree adjusted for the population density (residents/mi^2^), the percentage of individuals <5 years of age and >64 years of age, county, and the centroid of the census tract. See Tables S2 and S3 in Supporting Information S1 for details on the effect estimates in (b) and (c), respectively.

*K*‐means clustering was utilized to characterize the typical tracts' occupational and environmental exposures. The optimal number of clusters was *k* = 3 and the location of these clusters is mapped in Figure 4a. The average values of each occupational and environmental indicator for the clusters are found on Figure S6 in Supporting Information S1. Cluster 1 (*n* = 849 tracts) typically had the lowest value of each occupational indicator on average, but average environmental exposures, representing tracts with low occupational and medium environmental exposures. Tracts in Cluster 1 were generally in areas around larger cities, although this cluster included some rural areas in Northern Michigan and Southwest Michigan. Cluster 2 (*n* = 1,149 tracts) typically had high occupational exposures but low environmental exposures, representing occupationally—but not environmentally—burdened communities. These tracts were typically in rural areas. Cluster 3 (*n* = 738 tracts) had the highest values of the occupational and environmental indicators on average, representing tracts with the cumulatively high occupational and environmental exposures. Generally, though not ubiquitously, tracts in Cluster 3 were near city centers, primarily in the Detroit, Flint, Lansing, and Grand Rapids metropolitan areas.

### 3.4 Association Between Marginalized Communities and Cumulatively High Occupational and Environmental Exposures

Using a gradient boosted regression tree model adjusted for the population density, the percentage of individuals <5 years of age and >64 years of age, county, and the centroid of the census tract, we observed that census tracts with a simultaneously high proportion of marginalized communities had significantly higher odds of being in Cluster 3 (high occupational and environmental exposures) compared to Clusters 1 and 2 (Figure 4b). Census tracts at the 90th percentile of racial and ethnic minority individuals, low‐income individuals, and those without a high school diploma had 3.38 (95% CI: 2.72–4.38) times higher odds of being in Cluster 3 compared to the 50th percentile, while tracts at the 10th percentile had 0.44 (95% CI: 0.35–0.54) times lower odds of being Cluster 3 compared to the 50th percentile (Table S2 in Supporting Information S1). Independently, census tracts with >90% of racial and ethnic minority individuals had 2.31 (95% CI: 1.78–3.03) times higher odds of being in Cluster 3 compared to those with <10% of racial and ethnic minority individuals (Figure 4c). Similarly, census tracts with >60% of low‐income individuals had 2.62 (95% CI: 2.14–3.15) times higher odds of being in Cluster 3 compared to those with <10%, and 1.62 (95% CI: 1.32–2.04) times higher odds among census tracts with 12%–15% of individuals without a high school diploma compared to those with <3% (Table S3 in Supporting Information S1).

### 3.5 Association Between Cumulatively High Occupational and Environmental Exposures and Historical Redlining

**Figure 5:**
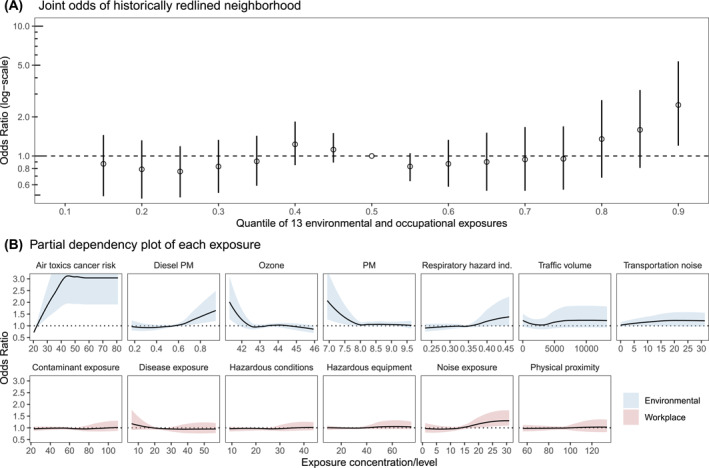
(a) Odds (95% confidence interval) of a census tract having been historically redlined (Grade D compared to Grades A–C) with increasing occupational and environmental exposures. Effect reflects the ratio in odds when the 13 exposure indicators (Environmental: air toxics cancer risk; diesel PM; ozone; PM_2.5_; respiratory hazard index; traffic volume; and transportation noise indicator. Occupational: average frequency of days exposed to contaminants, disease/infections hazardous conditions, hazardous equipment, the prevalence of hazardous workplace noise exposure, and frequency of close physical proximity with other workers) are fixed at a specific quantile (ranging from 0.15 to 0.9), as compared to when the factors are fixed at their median value. (b) Univariate relationships between the 13 environmental (shaded in blue) and occupational (shaded in red) exposures and the odds (95% confidence interval) of a census tract having been historically redlined (Grade D compared to Grades A–C). The dotted lines represent the null effect. Odds ratios modeled using a gradient boosted regression tree adjusted for the percentage of low‐income individuals and individuals without a high school diploma. See Table S6 in Supporting Information S1 for details on the effect estimates in (a).

D‐graded census tracts across Michigan generally had the highest proportion of marginalized individuals, highest occupational exposures, and highest environmental exposures (Table S4 in Supporting Information S1). Mixed‐effects conditional autoregressive models showed that D‐graded tracts did not have statistically significantly higher risk of physical proximity to other workers, disease/infection exposure, ozone, respiratory hazard index, nor transportation noise; all other occupational and environmental exposures displayed a significantly positively association with D‐graded tracts (Table S5 in Supporting Information S1). A simultaneous increase to the 90th percentile in all 13 occupational and environmental exposures was significantly associated with 2.47 (95% CI: 1.20–5.36) times higher odds of a census tract having been historically redlined compared to the 50th percentile (Figure 5a), adjusting for the percentage of low‐income individuals and individuals without a high school diploma. Meanwhile, setting all of the exposure to their 10th percentile did not display a significant association (OR = 1.10, 95% CI: 0.56–1.72) (Table S6 in Supporting Information S1). Partial dependency plots showed positive, independent associations between cancer risk from air toxics, diesel particulate matter, respiratory hazard index, traffic volume, transportation noise, and occupational noise exposure with higher odds of a census tract having been historically redlined in Michigan (Figure 5b).

### 3.6 Case Study of MiEJScreen With Occupational Exposures

**Figure 6:**
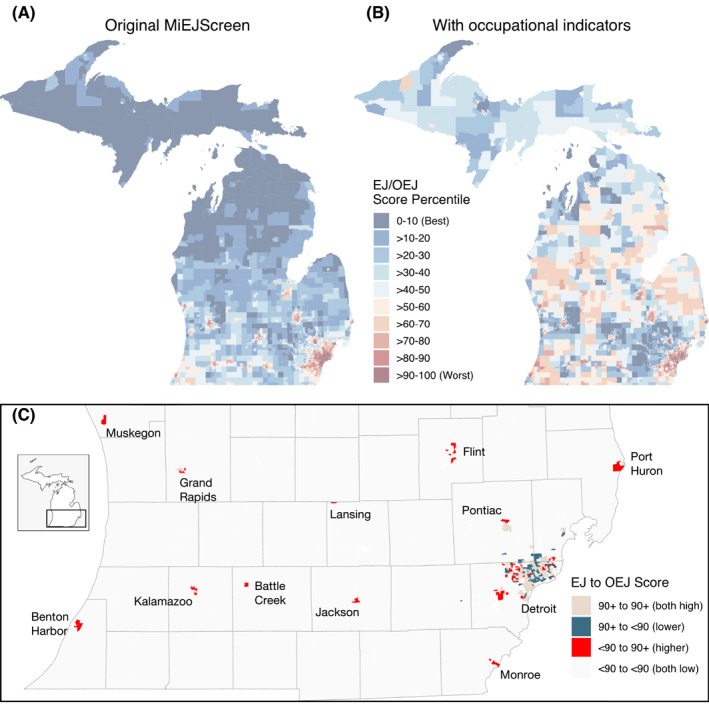
(a) Map of the overall environmental justice (EJ) score (0–100, with 100 being the most burdened communities) for each census tract in Michigan from the draft MiEJScreen. (b) Map of the newly calculated, overall occupational/environmental justice (OEJ) score for each census tract in Michigan by incorporating six occupational exposure indicators (average frequency of days exposed to contaminants, hazardous equipment, hazardous conditions, disease/infections, and physical proximity with other workers, and the prevalence of hazardous workplace noise exposure) into the draft MiEJScreen. (c) Change in overall score between the original MiEJScreen and after inclusion of occupational exposure indicators: both scores above 90 (colored in tan); original EJ score above 90 but OEJ score below 90 (colored in blue); original EJ score below 90 but OEJ score above 90 (colored in red); and both scores below 90 (colored in gray). Note that all other census tracts in Michigan not shown in (c) had both scores below 90. Cities with a new OEJ score above 90 are labeled.

Figure 6a displays the originally drafted MiEJScreen, without inclusion of any occupational indicators. Figure 6b displays how the MiEJScreen would look like if the six occupational exposures in this study were incorporated into the MiEJScreen. Figure S7 in Supporting Information S1 provides a more granular look between the MiEJScreen without and with occupational indicators in Detroit, MI. Of the 276 census tracts originally identified to have an overall EJ score ≥90 (the most overburdened EJ communities), *n* = 90 of these tracts (33%) would have scores <90 when incorporating occupational exposures and be replaced by 90 different tracts across Michigan. The majority of these newly identified 90 tracts would be from Detroit (*n* = 43), Flint (*n* = 9), and Muskegon (*n* = 6) metropolitan areas (Figure 5c), accounting for around 255,000 residents. Importantly, 30 of these 90 tracts would be identified across nine new cities which did not have any tracts with an overall score ≥90 in the original MiEJScreen (Flint, Port Huron, Lansing, Monroe, Jackson, Battle Creek, Kalamazoo, Benton Harbor, and Muskegon). Even after inclusion of the occupational exposures, 186 of the original 276 tracts with an original, overall EJ score ≥90 would still have a score ≥90, with the vast majority continuing to be identified in Wayne County (158/186, or 85% of tracts).

Figure S8 in Supporting Information S1 displays a sensitivity analysis in the overall EJ score when including the occupational indicators in the “Population Characteristics” subscore rather than the “Environmental Conditions” subscore (as displayed in Figure 6c). A sensitivity analysis of calculating the overall EJ score. While the main approach identified 90 new census tracts with an overall score ≥90, inclusion of the occupational indicators in the “Population Characteristics” subscore would identify 20 new census tracts with an overall score ≥90. The main approach identified new census tracts all across cities in Southern Michigan (Figure S8A in Supporting Information S1); the sensitivity approach only identified new census tracts around the Detroit metropolitan area (Figure S8B in Supporting Information S1).

## 4 Discussion

This place‐based study of environmental and occupational exposures investigated cumulative environmental and occupational injustices in Michigan. The findings demonstrated that cumulatively high environmental and occupational exposures likely exist, especially in urban centers of Michigan. Marginalized communities, particularly racial and ethnic minority individuals, may be the most burdened by these cumulative impacts. Historical, racialized residential segregation may partially explain why this cumulative inequity is observed in urban contexts. The findings suggest that ignoring workplace exposures in environmental justice assessments may under‐characterize injustices occurring within some communities, and some of the most burdened communities may not be identified if exposures in the workplace are not considered.

Independent of ambient, environmental exposures, this study provides evidence of occupational justice issues across Michigan. While our findings indicate that inequities to environmental exposures may be wider compared to occupational exposures, regulations that control workplace hazards are orders of magnitude higher than regulations that protect the public from ambient pollution (Environmental Protection Agency, 2013; Occupational Safety and Health Administration, 1999). This can help explain why health disparities are observed among racial and ethnic minority individuals in the U.S. For example, our findings of increased exposure to workplace noise, chemical contaminants, hazardous equipment use and hazardous work conditions highlight disparate workplace conditions for racial and ethnic minority individuals (Stephan‐Recaido et al., 2024). This is consistent with studies that have shown elevated workplace injury risk of Black and Hispanic workers (Sabbath et al., 2017; Seabury et al., 2017), injuries which may lead to disabilities and diseases which perpetuate systemic economic deprivation among these marginalized communities. Furthermore, the observed disproportionate exposure to disease/infection and closer physical proximity to other workers among racial and ethnic minority workers may help explain why racial and ethnic minority communities disproportionately felt the effects of the COVID‐19 pandemic (Asfaw, 2022; McClure et al., 2020).

When considering both occupational and environmental exposures together, results from the *k*‐means clustering algorithm supported the notion of cumulatively high exposures to both sources. These communities may require the most immediate, targeted interventions and changes in policies, particularly if low‐income individuals and individual with low educational attainment make up majority of the demographic makeup. This is especially relevant when considering occupational exposures alongside environmental exposures, as an individual's occupation is often linked to their socioeconomic status, and thus, their power and resources to ameliorate the adverse health effects of these exposures (Link & Phelan, 1995). Our analysis of the percentage of marginalized populations highlighted this point; racial and ethnic minority individuals, low‐income individuals, and individuals without a high school diploma disproportionately live in communities with cumulatively high occupational and environmental burdens. These communities could not only be disproportionately exposed in their home environments, but also without reprieve when going to work, likely exacerbating racial and ethnic minority social vulnerability to environmental toxicants (Gee & Payne‐Sturges, 2004). This notion is crucial to consider in cohort‐based studies; for example, a 2023 study investigating the relationship between transportation noise and insulin resistance/diabetes found that the effects of transportation noise on insulin resistance/diabetes was higher among residents of low SES communities that high SES communities (Letellier et al., 2023). This effect modification may be driven by cumulative exposure to noise (along with other occupational hazards that may cause stress) at work beyond their residential/recreational exposures.

Racialized residential segregation via historical policies such as redlining may explain why cumulative occupational and environmental injustices were observed in urban contexts. Our findings suggest that historically redlined neighborhoods in Michigan are overburdened by both environmental and occupational exposures, particularly cancer risk from air toxics in the community setting and occupational noise in the workplace setting. This is corroborated with previous studies which have found that historically redlined neighborhoods are more proximate to oil/gas well and power plant siting (Cushing et al., 2022; Gonzalez et al., 2022), suggesting that areas near these neighborhoods tend to be more industrial and could be shaping elevated exposures in the workplace. These findings provide further evidence for the conceptual framework guiding this study (Figure 1); urban residential segregation may be an important social mechanism that could explain why cumulative inequities exist in Michigan cities and likely throughout the United States.

Our case study of the MiEJScreen demonstrated the importance of considering occupational exposures in such tools related to cumulative environmental impacts. Even after including the occupational indicators, most census tracts in cities remain as EJ hotspots. This does not suggest that incorporating occupational indicators provides limited new knowledge; rather, it highlights how cumulative environmental and occupational exposures may be burdening EJ communities in cities across Michigan. This suggests that even if the environment is cleaned up in these communities, residents may still get sick if their workplaces aren't also prioritized. More crucially, many new census tracts in cities across Southern Michigan were identified as potential EJ hotspots once occupational exposures are incorporated, suggesting that other burdened communities may not be receiving the proper resources and interventions to prevent and reduce adverse health outcomes within the current status quo of considering ambient, environmental exposures only.

### 4.1 Limitations and Strengths

Identification of EJ hotspots using occupational indicators alongside environmental indicators is greatly susceptible to the choice of indicators. While the six occupational indicators used in our analysis were chosen because of the commonality of these six hazards across multiple industries and occupations and across multiple hazard domains (i.e., physical, chemical, safety, and biological hazards), development of occupational indicators to other workplace hazards may identify new EJ hotspots across Michigan and other states. Further indicators can be created from different JEMs using the same method, such as the percentage of workers exposed to carcinogenic or asthmogenic chemicals for every census tract. The percentage of outdoor workers who may exposed to hazardous levels of heat is an example of an indicator that may be more appropriate for state‐specific EJ screening tools in the Southern US. Further, our analysis only accounted for environmental exposures in the EJScreen, not the entire exposome of an individual. This leaves off other important contaminants and pollutants, such as PFAS. As a result, the findings of this study highlight inequities in cumulative occupational and environmental injustices among the set of exposures considered here, and not the true reality of all exposures; regardless, this study provides a starting point for an important topic that remains underexamined.

It is important to note that five of the six occupational indicators used in this study were developed on relatively crude underlying data. The five frequency occupational indicators used O*NET data, which relies on self‐reported perceptions of workplace hazards. While the utility of the O*NET cannot be disregarded, developing spatiotemporal indicators using subjective data can make it difficult to ascertain the validity of these indicators. A previous study by some members of our study team demonstrated that misperception of noise exposure during work is relatively common, although no substantial differences in the direction of this misperception were observed (i.e., there was nearly the same proportion of workers underestimating exposures as there were overestimating exposures) (Shkembi et al., 2023). This non‐differential exposure misclassification suggests that using self‐reported data to construct the occupational indicators in this study may bias the estimates in either direction, although knowledge of workplace hazard misperceptions is severely limited. The noise occupational indicator, however, used data from more than 1 million noise measurements taken during work across all industries and occupations. Future development of occupational indicators for inclusion in cumulative impacts assessment should also seek to use objective measurements of workplace hazards, rather than relying solely on self‐reported perceptions. Future research should also attempt to determine the validity of these occupational indicators.

The choice of weights to each indicator when constructing an overall EJ score may lead to different conclusions of how the occupational indicators influence the identification of EJ hotspots in Michigan. This is a limitation of simplifying complex and overlapping exposures and population characteristics into a single score. Indeed, when the occupational indicators were incorporated into the “Population Characteristics” subscore, different census tracts were identified as hotspots. We believe that it is ideal to include the occupational indictors in the “Environmental Conditions” category and weight the occupational indicators by 1.5 such that they are equally weighted to the “Environmental Exposures” and “Environmental Effects” indicators. However, other developers of EJ screening tools may wish to consider how important occupational exposure are for their purpose of assessing cumulative impacts, and either up‐ or down‐weight the occupational indicators accordingly or include occupational indicators in an entirely different category.

This study performed a limited analysis of racial and ethnic inequities by not breaking down analyses by racial and ethnic groups. This is because this study was not motivated by inequities among any particular racial and ethnic minority group, although it is important to acknowledge that cumulative environmental and occupational inequities likely impact different racial and ethnic minority groups differently.

This study's analytical strategy is susceptible to the modifiable areal unit problem. While these estimates are not point‐based measures, the construction of these measures is based on aggregated estimates of employment count for an arbitrary administrative boundary—the census tract. The employment count estimates from the US Census ACS are the most important strength of this study's approach to developing community‐level estimates of workplace exposure. These estimates are based on the resident's home location, not the place of their employment. This allows us to properly merge environmental exposure measures for the same census tracts where the resident lives, without concern of a mismatch in occupational and environmental exposures.

### 4.2 Implications

Due to the separation of responsibilities between environmental and occupational governmental agencies, currently proposed cumulative impacts policy through the EPA and state‐level environmental departments would likely be limited to considering and controlling environmental exposures only. For workers in traditional, high hazard industries, the NIOSH Total Worker Health (TWH) framework may represent an alternative avenue to reduce cumulative exposures through the workplace. The TWH framework acknowledges that hazards in and out of the workplace contribute to injuries and illnesses at work (Schill & Chosewood, 2013), a framework similar to cumulative environmental impacts. With this in mind, employers have the opportunity to understand the full scope of their workers' exposures not only during work, but also at home, through sampling of workers' environmental exposures and by providing resources to reduce exposures outside of the workplace. Unfortunately, non‐traditionally exposed workers, such as office workers who may be exposed to dozens of chemicals in buildings (Young et al., 2021) and face ergonomic issues, or exposures of precariously employed workers, such as migrant farmworkers (Castillo et al., 2021), are frequently overlooked, under‐sampled, and don't represent a plausible avenue for tackling cumulative exposures. However, advancements in small, wearable technology (from as complex as smart watches (Neitzel et al., 2022) to as simple as silicone wristbands (Samon et al., 2022)) have made sampling for pollutants widespread and affordable and may present an avenue for collecting information on cumulative exposures.

It's important to note that while this study highlights that cumulative environmental and occupational exposures likely exist, other potential sources of exposure, such as consumer products or indoor pollution, may also cumulatively impact marginalized communities (Adamkiewicz et al., 2011; McDonald et al., 2022). One study found poor levels of cumulative outdoor and indoor air quality in industrial Richmond, California (Brody et al., 2009). On the role of beauty justice within environmental justice, marginalized communities who are disproportionately impacted by beauty products (Zota & Shamasunder, 2017) may also be face cumulative exposures by working in the beauty industry, such as Black hair‐salon and Vietnamese nail‐salon workers (Adewumi‐Gunn et al., 2018; Quach et al., 2011). As this study made fact of the current place‐based assessments of environmental pollution by incorporating place‐based estimates of occupational exposures, future studies should consider developing spatial indicators of indoor air quality and prevalence of toxic consumer product use. This is particularly important for children, who like adult workers, typically spend a majority of their day away from their home neighborhood and may be additionally exposed to poor indoor air quality at school and from buses (Brink et al., 2021; Pedde et al., 2023).

## 5 Conclusion

Marginalized communities in Michigan disproportionately suffer cumulatively high exposures both in and out of the workplace, and historically racist housing discrimination may have contributed to this cumulative inequity. Ignoring occupational exposures in cumulative environmental impact assessments may overlook important EJ hotspots. Further work is needed to better characterize the full scope of occupational hazards facing workers today.

## Conflict of Interest

The authors declare no conflicts of interest relevant to this study.

## Supporting information

Supporting Information S1

## Figures and Tables

**Figure A1:**
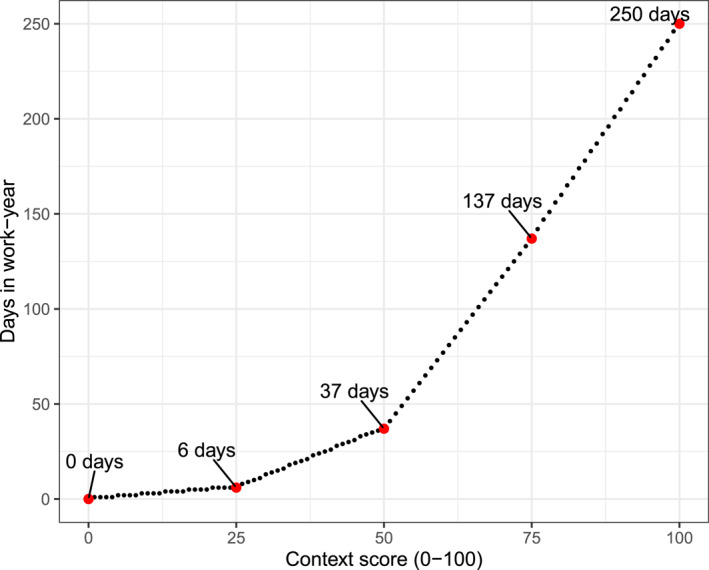
Constructed relationship between context scores and days exposed in a standard work year (250 days). O*NET scores range from 0—100 (0: never; 25: Once a year or more but not more than every month; 50: Once a month or more but not every week; 75: Once a week or more but not every day; and 100: Every day). Assuming a 250 day, standard work‐year, context scores were reassigned to equivalent work days (Context score of 0: 0 work days; Context score of 25: midpoint of 1–12 work days, or 6 days; Context score of 50: midpoint of 12–50 work days, or 31 days, plus the 6 previous work days for a total of 37 days; Context score of 75: midpoint of 50 and 150 work days, or 100 days, plus the previous 37 work days for a total of 137 days; and finally, Context score of 100: 250 work days).

**Figure A2:**
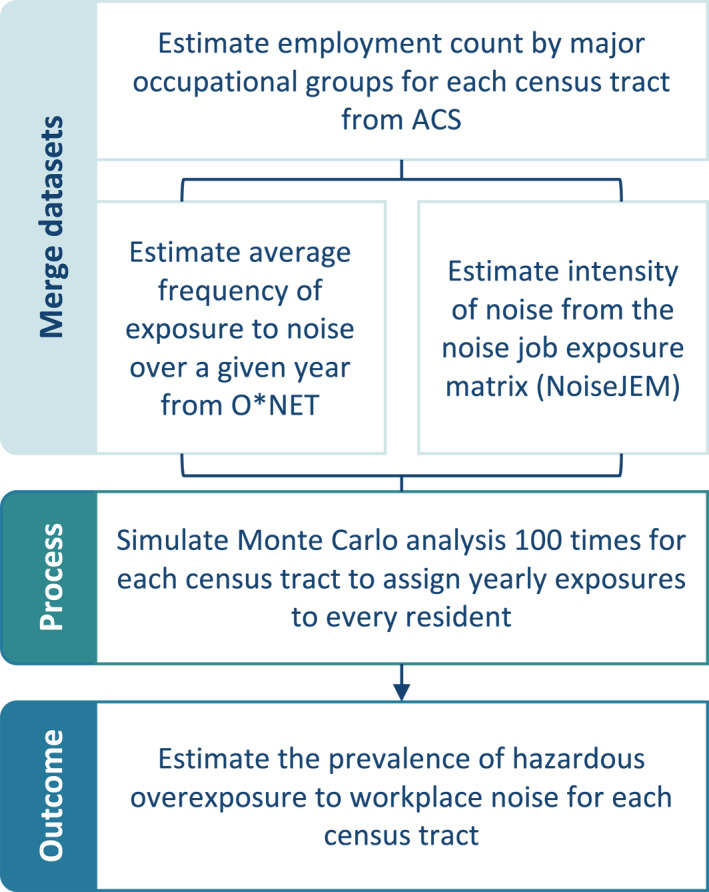
Monte Carlo workflow to estimate prevalence overexposure to workplace noise by census tract.

## Data Availability

All data came from publicly available sources: (a) the 5‐year, 2015–2019 US Census ACS (U.S. Census Bureau, 2020), (b) the Department of Labor Occupational Information Network (U.S. Department of Labor, 2019), (c) a US/Canada noise job exposure matrix (NoiseJEM) (University of Michigan, 2020), (d) the EPA EJScreen (United States Environmental Protection Agency, 2020), (e) historical redlining shapefiles from the Home Owners’ Loan Corporation from the Mapping Inequality Project (Nelson et al., 2017), and (f) the MiEJScreen (Office of the Environmental Justice Public Advocate, 2022).

## Appendix A Construction of Occupational Indicators

Detailed information on the construction of the occupational indicators has been previously published by our study team (Shkembi, Zelner, et al., 2024). Of the six occupational hazard indicators constructed, five of the indicators were frequency indicators (i.e., the average number of days spent exposed over a given year). The sixth indicator was the estimated percentage of workers exposed to high noise over a given year, which utilized both frequency and intensity information. To create the six occupational hazard indicators, we leveraged three existing data resources. A brief summary of these resources is provided below.

1. US Census American Community Survey (ACS)—we utilized 5‐year ACS from 2015 to 2019 to gather total employment count for each US census tract and breakdowns by major occupational groups for 2017. There are 22 major non‐military groups. The ACS follows the Census Occupation Code structure for classifying occupational groups and can be linked to the Bureau of Labor Statistics (BLS) Standard Occupational Classification (SOC) structure. The data was collected using the “tidycensus” R package (Walker & Herman, 2022).
2. Department of Labor (DoL) O*NET—we accessed DoL O*NET Physical Work Conditions data on the frequency of chemical, physical, and biological hazards for each major occupation code. *Chemical hazards* were assessed through exposure to pollutants (“How often does this job require working exposed to contaminants (such as pollutants, gases, dust or odors)?”). *Physical hazards* were assessed through exposure to hazardous equipment (“How often does this job require exposure to hazardous equipment?”), hazardous conditions (“How often does this job require exposure to hazardous conditions?”), and noise exposure (“How often does this job require working exposed to sounds and noise levels that are distracting or uncomfortable?”). *Biological hazards* were assessed through exposure to disease/infections (“How often does this job require exposure to disease/infections?”) and physical proximity to other workers (a combination of “To what extent does this job require the worker to perform job tasks in close physical proximity to other people?” and “How often do you have to have face‐to‐face discussions with individuals or teams in this job?”), which, along with exposure to noise, are risk factors for COVID‐19 and other infectious disease transmission in the workplace (Shkembi & Neitzel, 2022; Zhang, 2021). Each frequency is presented on a continuous scale of 0–100, referred to herein as “Context” scores. The scale is broken down into the following descriptors: 0 is “Never”, 25 is “Once a year or more but not every month”, 50 is “Once a month or more but not every week, 75 is “Once a week or more but not every day,” and 100 is “Every day.” The data followed the O*NET‐SOC structure, which can be cross‐walked with the BLS SOC structure.
3. NoiseJEM—we used the US/Canada noise job exposure matrix (NoiseJEM) to estimate workplace noise intensity by job category. The JEM contains mean, median, minimum, maximum, and standard deviation 8‐hr time‐weighted averages (TWA‐8h) levels measured according to the criteria of the National Institute for Occupational Safety and Health (NIOSH) and Occupational Safety and Health Administration (OSHA) from 1963 to 2015. Exposure estimates were available across all jobs and industries within the JEM. Classification of job titles followed the BLS SOC structure. The BLS SOC structure was linked with the Census Occupation Code structure. Measurements using OSHA Permissible Exposure Limit samples were used for analysis in this study.

Context scores from O*NET are difficult to interpret; further, a one‐unit change in a context score is not the same as a one‐unit change the days within a year, which is what context scores are measuring. To make this measure easier to interpret, we translated context scores into day‐equivalents (for a typical 250‐day work‐year) using the relationship in Figure A1.

O*NET provides data at the detailed occupation level, so we estimated average days exposed to a particular hazard for each major occupational group (see Table A1) and referred to this as the mean frequency score, *F*_*i*_, for a given major occupation group *i*.

**Table A1:** Average Days Exposed by O*NET Hazard and Major Non‐Military Occupation Group

Major SOC	Physical proximity	Chemical contaminant	Hazardous equipment	Hazardous conditions	Disease/infection
11‐0000	28.2	21.3	13	16	5.8
13‐0000	19.9	13.3	3.7	1.9	4.8
15‐0000	8	2.9	1.1	1.2	2.1
17‐0000	4.3	27.4	23.9	21.9	1.9
19‐0000	24.7	36.3	11.8	28	10.1
21‐0000	83.9	10.1	0.7	1.2	36.9
23‐0000	18.4	2.6	0.4	0.6	2
25‐0000	67.3	9.7	2.2	4	11.6
27‐0000	72	11.6	6.6	2.7	2.4
29‐0000	215	44.7	8.5	19.2	167
31‐0000	188	54.5	3.4	18.2	152
33‐0000	163	86	43.8	29.5	51
35‐0000	166	15.4	9.9	7.1	6.1
37‐0000	45.6	130	79.9	56.2	14.6
39‐0000	173	44.1	2.5	13.2	26
41‐0000	93	21.5	3.4	2.1	2.3
43‐0000	51.5	20.7	6.1	2.3	9.5
45‐0000	63.9	96.6	57.1	10.7	6.9
47‐0000	152	148	128	62.9	6.2
49‐0000	102	137	112	78.3	3.7
51‐0000	52.1	138	87	54	3.9
53‐0000	98.3	135	76	50	12

*Note.* Major SOC refers to major occupation groups specified by the Bureau of Labor Statistics Standard Occupational Code classification system. 11‐0000 Management Occupations; 13‐0000 Business and Financial Operations Occupations; 15‐0000; Computer and Mathematical Occupations; 17‐0000 Architecture and Engineering Occupations; 19‐0000 Life, Physical, and Social Science Occupations; 21‐0000 Community and Social Service Occupations; 23‐0000 Legal Occupations; 25‐0000 Educational Instruction and Library Occupations; 27‐0000 Arts, Design, Entertainment, Sports, and Media Occupations; 29‐0000 Healthcare Practitioners and Technical Occupations; 31‐0000 Healthcare Support Occupations; 33‐0000 Protective Service Occupations; 35‐0000 Food Preparation and Serving Related Occupations; 37‐0000 Building and Grounds Cleaning and Maintenance Occupations; 39‐0000 Personal Care and Service Occupations; 41‐0000 Sales and Related Occupations; 43‐0000 Office and Administrative Support Occupations; 45‐0000 Farming, Fishing, and Forestry Occupations; 47‐0000 Construction and Extraction Occupations; 49‐0000 Installation, Maintenance, and Repair Occupations; 51‐0000 Production Occupations; 53‐0000 Transportation and Material Moving Occupations.

This information was then joined with employment count estimates of the major occupation groups by census tract. The employment counts were converted to proportions within a census tract, and a *weighted* frequency score for each major occupation group within a census tract was created by multiplying the proportion of those employed by the mean frequency scores. These weighted frequency scores were then summed within a given census tract to assign a single, summed weighted frequency score to that tract for each of the occupational hazards. The construction of the weighted frequency score for the frequency of an occupational exposure, *O*, for a given census tract, *i*, can be described by the equation below:

$$O_{i}=\sum_{j=1}^{22}p_{ij}\cdot F_{j}$$

where *p*_*ij*_ denotes the proportion of workers belonging to major occupation group *j* in census tract *i*. *F*_*j*_ denotes the mean frequency score for major occupation group *j* (*j* = 1, 2, 3, …, 22; note there are 22 major non‐military occupation groups). For example, imagine a census tract with 50% of its working population in service occupations, and the other 50% in farming, fishing, and forestry. We know from O*NET that the mean frequency scores for exposure to chemical contaminants for these major occupation groups is 20 and 60 days, respectively. We can then create a summed, weighted frequency score for this tract as follows: (0.5*20) + (0.5*60) = 40 days. This process can then be repeated for each census tract and each occupational hazard class. Specifically for the creation of the physical proximity indicator, *F*_*j*_ was adjusted by multiplying the frequency of face‐to‐face discussions by an indicator of the physical proximity question (1: >62.5, or halfway between “Slightly close” and “At arm's length”; 0: ≤62.5).

Next, we created the prevalence of exposure to hazardous noise indicator. Figure A2 provides an illustrative overview of the process. Using posterior distributions of each major occupation group from a hierarchical Bayesian imputation analysis of NoiseJEM (see Table A2) (Roberts et al., 2018) we sampled noise exposures equal to the number of workers within a major occupation group in a given census tract 100 times using a Monte Carlo simulation approach. Using these simulated exposures, we estimated the maximum allowable exposure time in the simulated workplace exposures over a given year under the assumptions that any given individual is exposed to the same noise levels every day, and that they work a typical 2,000‐hr (i.e., 250 8‐hr days) work‐year. The maximum allowable exposure time can be calculated using the following equation:

$$T_{\text{allowed}}=\frac{2,000}{2^{\frac{\left(TWA-85\right)}{5}}}$$

where exchange rate is 5 dB and an occupational exposure limit of 85 dBA (to which overexposure is associated with increased risk of hearing loss).

It is worth noting that while OSHA PEL noise measurements were used in this analysis, we used the occupational exposure limit (85 dBA) associated with the OSHA Action Level (AL) to determine overexposure as the OSHA PEL is not sufficiently protective of workers, and is less protective than the OSHA AL. Using the mean frequency score *F*_*i*_ for a given major occupation group, we converted the average days exposed to uncomfortable noise levels to hours (by multiplying days by 8 hr) to estimate the time exposed to hazardous noise levels for each major occupation group (see Table A2). Dose can then be estimated for any worker as a ratio of time exposed (assumed to be a standard 8‐hr shift for each individual) to time allowed, such that $\text{Dose}=100\times \frac{T_{\text{exposed}}}{T_{\text{allowed}}}$. From there, we estimated the percent of workers exposed to hazardous levels of noise over a typical work year for each census tract. We considered Dose >50% to be “hazardous” (which would equate to >80 dBA TWA‐8h) for two reasons: (a) an industrial hygienist would deem such exposures “unacceptable” in the workplace (American Industrial Hygiene Association Exposure Assessment Strategies Committee, 2015), and (b) Bayesian analyses of workplace exposure assessments have suggested that any average exposure within 50%–100% of the occupational exposure limit are highly likely to have a true exposure profile of Dose >100% (Hewett et al., 2006). Using the 100 Monte Carlo simulations, we calculated the mean percent of workers exposed to hazardous occupational noise and used this mean in subsequent analyses. Hearing protection device use was not incorporated in the Monte Carlo simulation as the extent of workplace exposures should be examine prior to considering any control (e.g., PPE, ventilation) to understand the full scope of the problem.

**Table A2:** Noise Characteristics for Monte Carlo Simulation by Major Non‐Military Occupation Group

Major SOC	Mean TWA (SD)	Hours exposed
11‐0000	81.8 (1.8)	314
13‐0000	82.7 (2.4)	220
15‐0000	80.9 (2.7)	155
17‐0000	80.7 (1.6)	392
19‐0000	82.8 (2.0)	283
21‐0000	80.7 (2.8)	294
23‐0000	78.4 (1.2)	152
25‐0000	84.0 (2.9)	214
27‐0000	82.1 (2.)	314
29‐0000	79.9 (1.8)	365
31‐0000	82.3 (2.9)	342
33‐0000	81.2 (1.8)	741
35‐0000	82.7 (1.7)	342
37‐0000	85.0 (2.5)	858
39‐0000	84.8 (1.9)	371
41‐0000	82.3 (2.1)	278
43‐0000	78.4 (1.2)	415
45‐0000	85.5 (2.0)	528
47‐0000	83.5 (0.9)	1,188
49‐0000	83.3 (1.2)	1,094
51‐0000	85.2 (0.7)	1,114
53‐0000	83.3 (0.9)	1,138

*Note.* Major SOC refers to major occupation groups specified by the Bureau of Labor Statistics Standard Occupational Code classification system. TWA = 8‐hr time weighted average from NoiseJEM. Hours exposed from O*NET over 2,000 standard workhours per year. 11‐0000 Management Occupations; 13‐0000 Business and Financial Operations Occupations; 15‐0000; Computer and Mathematical Occupations; 17‐0000 Architecture and Engineering Occupations; 19‐0000 Life, Physical, and Social Science Occupations; 21‐0000 Community and Social Service Occupations; 23‐0000 Legal Occupations; 25‐0000 Educational Instruction and Library Occupations; 27‐0000 Arts, Design, Entertainment, Sports, and Media Occupations; 29‐0000 Healthcare Practitioners and Technical Occupations; 31‐0000 Healthcare Support Occupations; 33‐0000 Protective Service Occupations; 35‐0000 Food Preparation and Serving Related Occupations; 37‐0000 Building and Grounds Cleaning and Maintenance Occupations; 39‐0000 Personal Care and Service Occupations; 41‐0000 Sales and Related Occupations; 43‐0000 Office and Administrative Support Occupations; 45‐0000 Farming, Fishing, and Forestry Occupations; 47‐0000 Construction and Extraction Occupations; 49‐0000 Installation, Maintenance, and Repair Occupations; 51‐0000 Production Occupations; 53‐0000 Transportation and Material Moving Occupations.
