# Supplementary material for: Racial and Ethnic Inequities to Cumulative Environmental and Occupational Impacts in Michigan
Source: Geohealth. 2025 Jun 20;9(6):e2025GH001482. doi: 10.1029/2025GH001482 (PMC12179436; doi:10.1029/2025GH001482)
Supplement: Supplementary file 1 — Supporting Information S1 [file GH2-9-e2025GH001482-s001.pdf]

AGU PUBLICATIONS

GeoHealth

Supporting Information for

Racial and Ethnic Inequities to Cumulative Environmental and Occupational Impacts in Michigan

Abas Shkembi, MS[1]; Sung Kyun Park, ScD[1,2]; Jon Zelner, PhD[2,3]; Richard Neitzel, PhD, CIH, FAIHA[1]

[1]Department of Environmental Health Sciences, University of Michigan School of Public Health, Ann Arbor, MI, USA

[2]Department of Epidemiology, University of Michigan School of Public Health, Ann Arbor, MI, USA

[3]Center for Social Epidemiology and Population Health, University of Michigan School of Public Health, Ann Arbor, MI, USA

Contents of this file

Figures S1 to S8
Tables S1 to S6

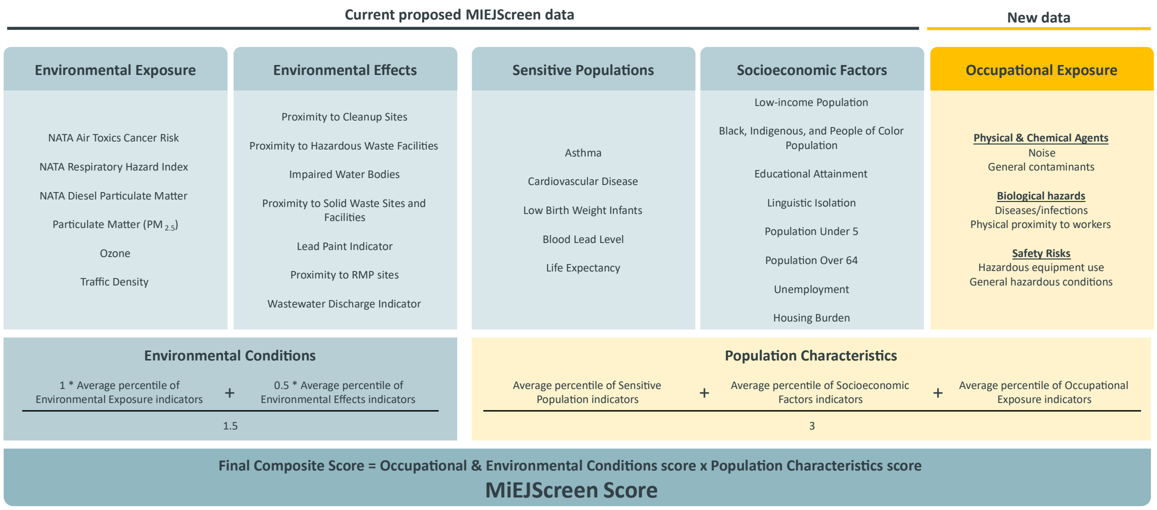

Figure S1. Construction of a sensitivity composite MiEJScreen score incorporating the six additional occupational exposure indicators, utilizing the same methodology used in the original MiEJScreen score (which currently does not include occupational indicators) but incorporating them in the Population Characteristics category rather than the Environmental Conditions category.

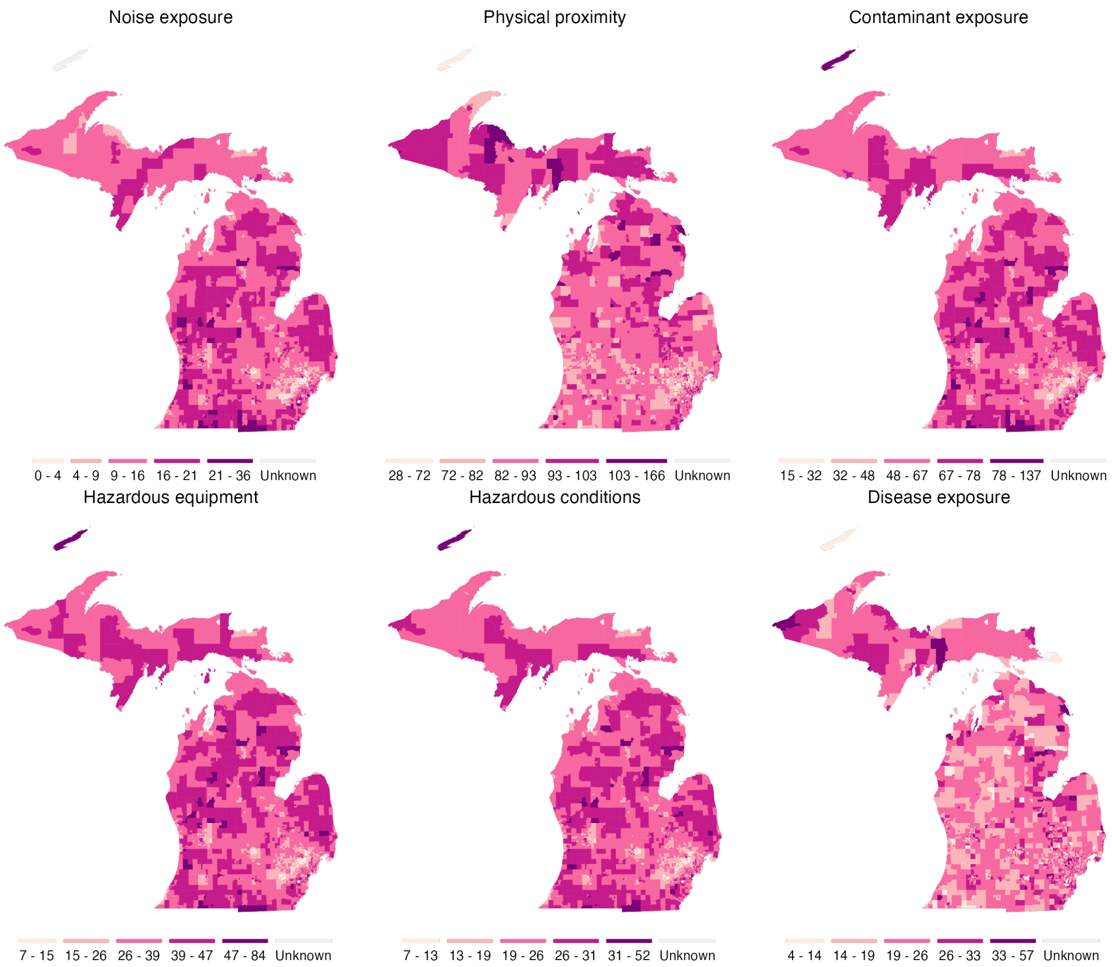

Figure S2. Prevalence of work-related noise exposure, average days in close physical proximity with other workers, average days exposed to chemical contaminants, average days using hazardous equipment, average days working in hazardous conditions, and average days exposed to disease/infection across Michigan census tract with working population >20 workers. The maps display each indicator's 0-10th percentile, 10-25th percentile, 25-75th percentile, 75-90th percentile, and 90-99th percentile.

(i)

	Hazardous noise	Physical proximity	Contaminant exposure	Hazardous equipment	Hazardous conditions	Disease/infection exposure	$PM_{2.5}$	Diesel PM	Ozone	Air toxics cancer risk	Respiratory hazard index	Traffic volume
Physical proximity	0.33											
Contaminant exposure	0.97	-0.10										
Hazardous equipment	0.94	0.34	0.98									
Hazardous conditions	0.94	0.37	0.98	0.98								
Disease/infection exposure	-0.25	0.39	-0.18	-0.27	-0.19							
$PM_{2.5}$	-0.09	-0.14	-0.14	-0.19	-0.17	0.01						
Diesel PM	-0.12	-0.11	-0.17	-0.24	-0.21	0	0.94					
Ozone	0.02	-0.18	0.30	-0.05	-0.04	-0.10	0.26	0.24				
Air toxics cancer risk	-0.11	-0.10	-0.17	-0.23	-0.21	0.02	0.90	0.94	0.30			
Respiratory hazard index	-0.12	-0.12	-0.17	-0.24	-0.22	0	0.92	0.97	0.25	0.97		
Traffic volume	-0.11	0	-0.17	-0.24	-0.22	0.02	0.67	0.79	0.15	0.78	0.78	
Transportation noise	-0.06	-0.09	-0.08	-0.09	-0.09	-0.04	0.17	0.23	0.12	0.21	0.23	0.36

(ii)

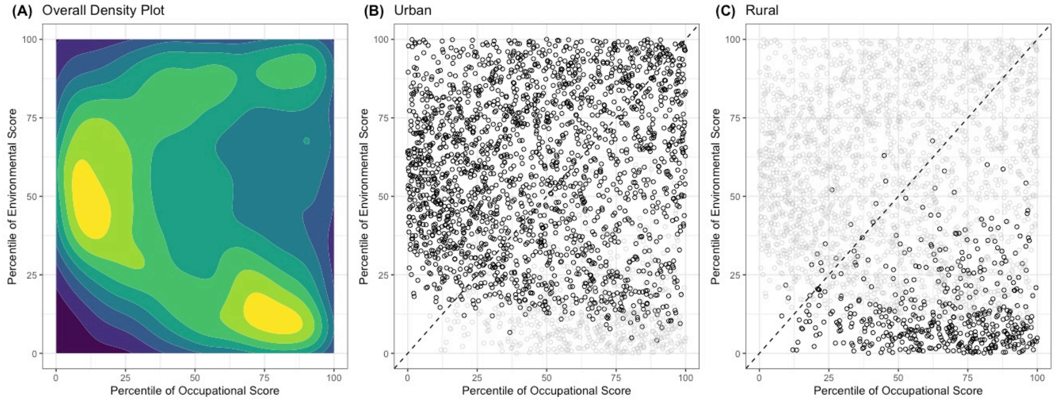

Figure S3. (i) Correlation matrix of occupational and environmental indicators. (ii) Relationship between average percentile of occupational indicators and average percentile of environmental indicators as a density plot, where more yellow colors indicate higher density (A), and by urban (B) and rural (C) census tracts.

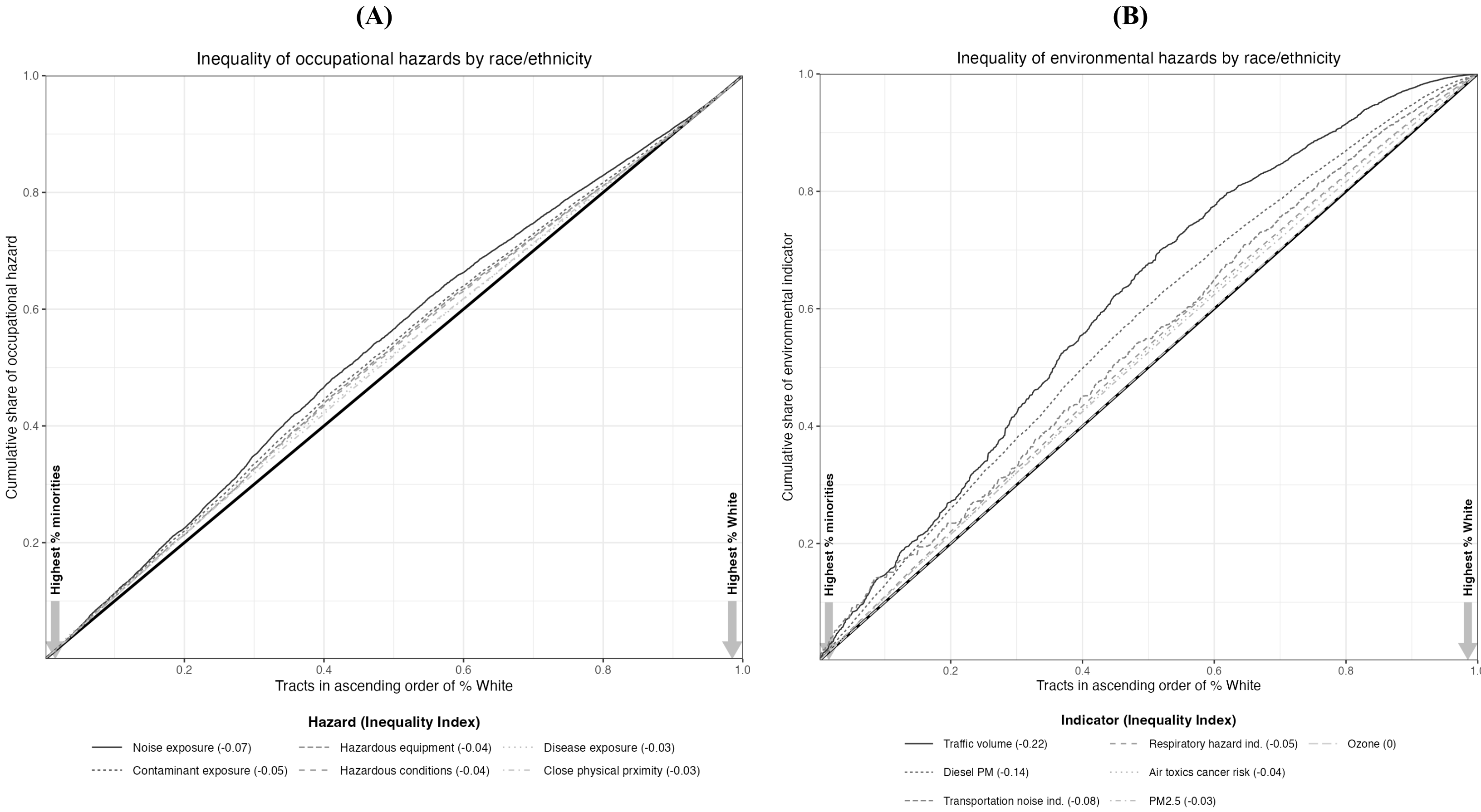

Figure S4. Inequality curves illustrating the distribution of (A) occupational hazard indicators and (B) environmental indicators across Michigan census tracts with tract-level percentage of non-Hispanic White individuals.

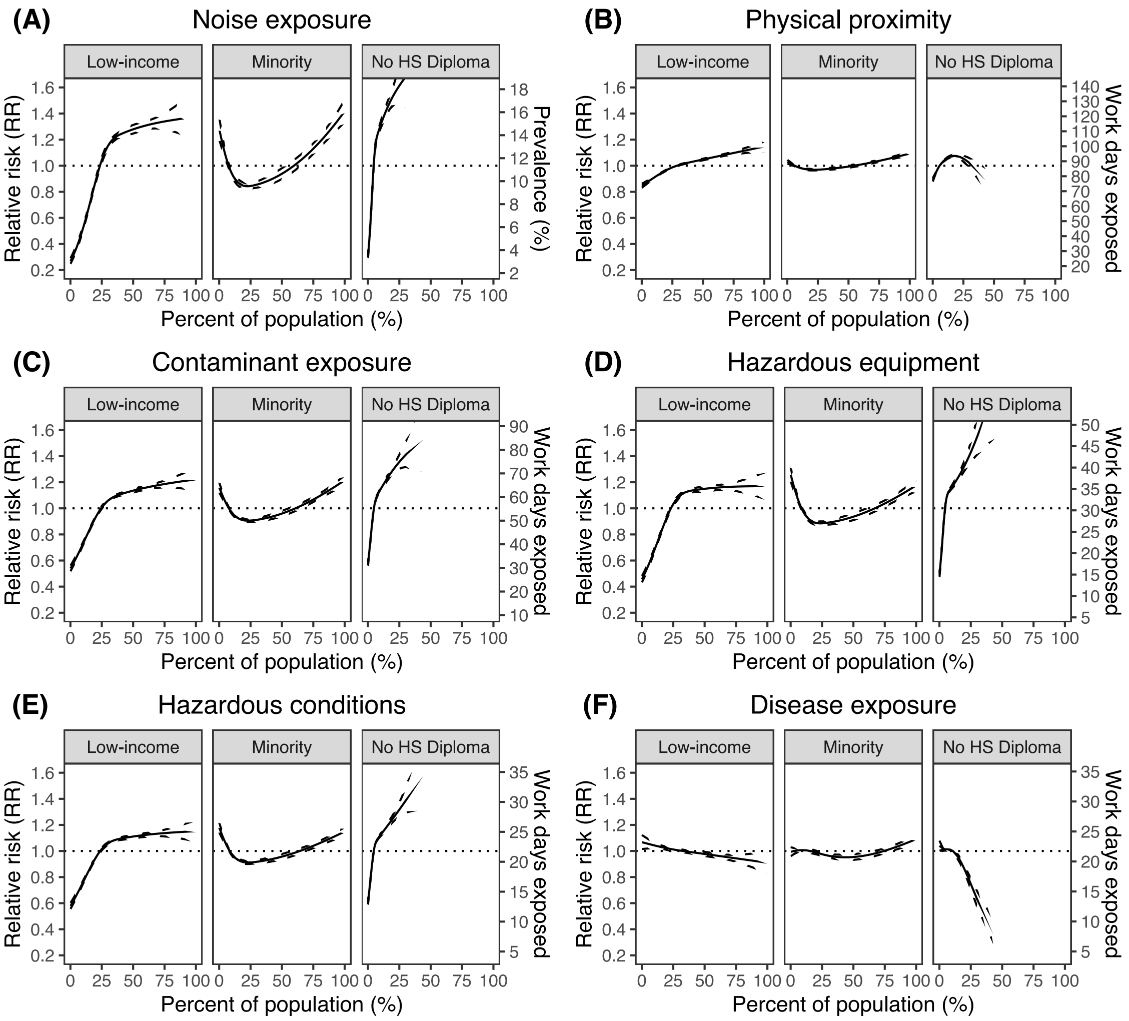

Figure S5. Relationship between percent of low-income (left), racial and ethnic minority groups (middle), or individuals without a high school diploma (right) population with (A) percent of workers exposed to hazardous noise, days exposed to (B) close physical proximity with other workers, (C) contaminants, (D) hazardous equipment, (E) hazardous workplace conditions, and (F) disease/infections using a natural cubic spline. Models were adjusted for resident population, urban versus rural status, and county.

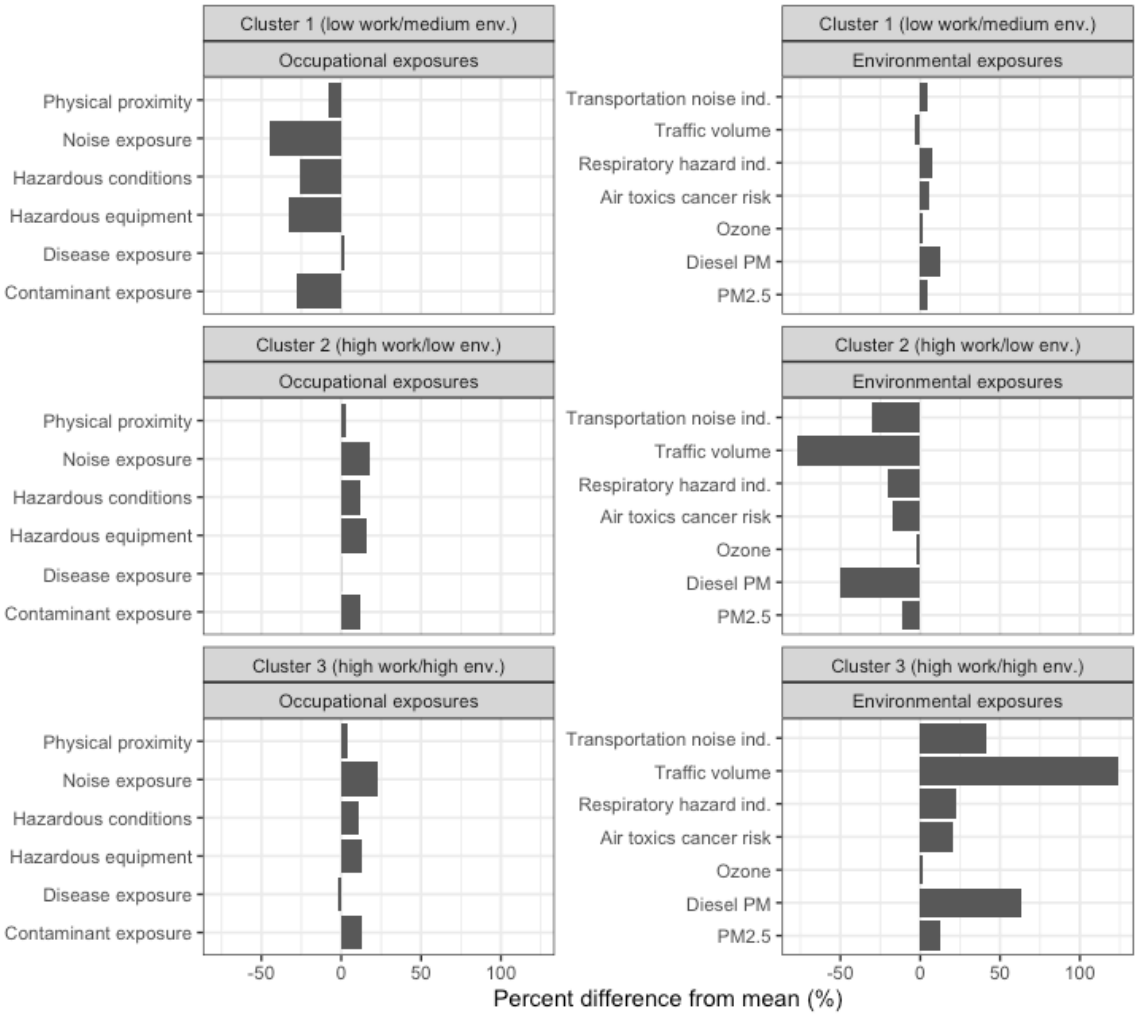

Figure S6. Results of k-means clustering algorithm using occupational and environmental exposures.

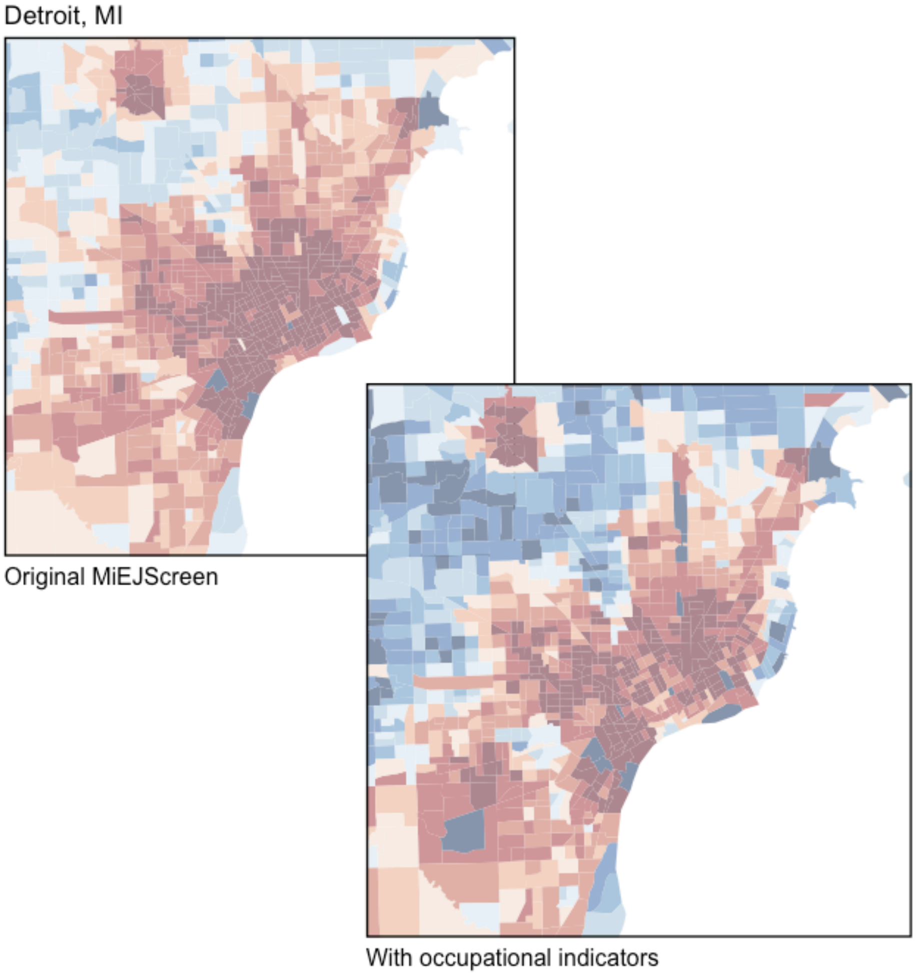

Figure S7. Comparison of census tracts in the Detroit, MI area in the original MiEJScreen (left) and after inclusion of occupational indicators into the MiEJScreen (right). Darker red indicates tracts with a higher EJ burden, while darker blue indicates lower EJ burden.

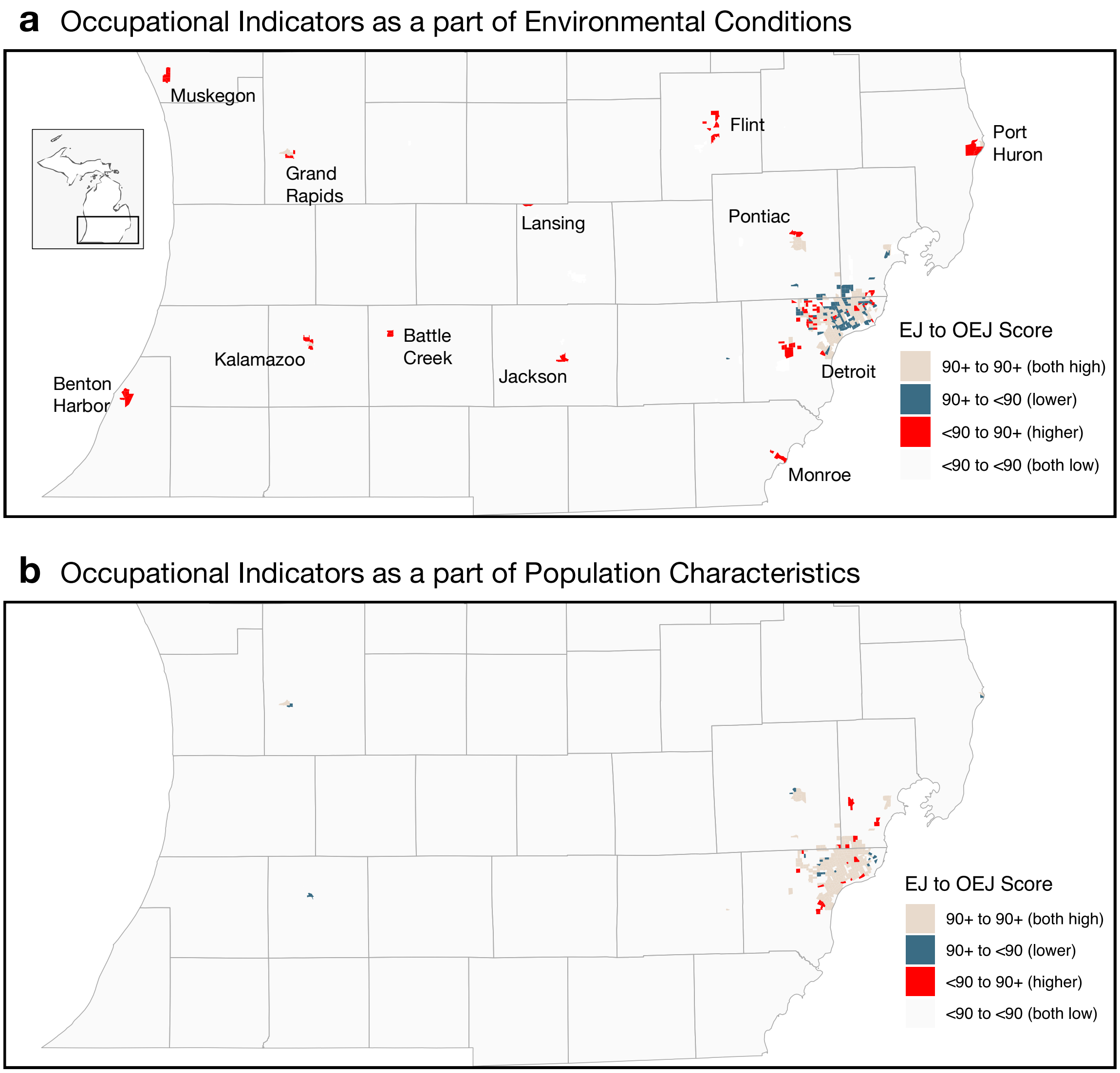

Figure S8. Sensitivity analysis of calculating a new OEJ score. (A) Change in overall score between the original MiEJScreen and after inclusion of occupational exposure indicators in the "Environmental Conditions" subscore, as displayed in Figure 6C of the main text. (B) Change in overall score between the original MiEJScreen and after inclusion of occupational exposure indicators in the "Population Characteristics" subscore. Legend: (1) both scores above 90 (colored in tan); (2) original EJ score above 90 but OEJ score below 90 (colored in blue); (3) original EJ score below 90 but OEJ score above 90 (colored in red); and (4) both scores below 90 (colored in grey). Note that all other census tracts in Michigan not shown in (C) had both scores below 90. Comparison of census tracts in the Detroit, MI area in the original MiEJScreen (left) and after inclusion of occupational indicators into the MiEJScreen (right). Darker red indicates tracts with a higher EJ burden, while darker blue indicates lower EJ burden.

Variable	Noise exposure	Physical proximity	Contaminant exposure	Hazardous equipment	Hazardous conditions	Disease/infection exposure
Percent of racial and ethnic minority groups						
0-10% (reference)	1	1	1	1	1	1
10-20%	0.98 (0.94, 1.02)	1.00 (0.99, 1.01)	0.98 (0.96, 1.00)	0.97 (0.94, 0.99)	0.97 (0.94 (0.99)	1.02 (0.99, 1.04)
20-30%	0.98 (0.92, 1.03)	1.00 (0.98, 1.02)	0.98 (0.95, 1.01)	0.96 (0.92, 0.99)	0.96 (0.93 (1.00)	0.99 (0.96, 1.03)
30-40%	1.03 (0.97, 1.10)	1.02 (1.00, 1.04)	1.01 (0.98, 1.05)	0.99 (0.95, 1.03)	0.99 (0.95, 1.03)	1.01 (0.96, 1.05)
40-50%	1.08 (1.00, 1.17)	1.00 (0.98, 1.02)	1.04 (1.00, 1.08)	1.03 (0.98, 1.08)	1.02 (0.97, 1.07)	1.00 (0.95, 1.05)
50-60%	1.17 (1.06, 1.28)	1.04 (1.01, 1.07)	1.08 (1.03, 1.13)	1.06 (0.99, 1.12)	1.04 (0.98, 1.11)	1.00 (0.93, 1.06)
60-70%	1.15 (1.05, 1.27)	1.02 (0.99, 1.05)	1.10 (1.05, 1.15)	1.07 (1.01, 1.14)	1.07 (1.00, 1.13)	1.03 (0.96, 1.10)
70-80%	1.24 (1.10, 1.40)	1.08 (1.04, 1.11)	1.11 (1.05, 1.15)	1.06 (0.98, 1.15)	1.05 (0.97, 1.13)	1.11 (1.03, 1.20)
80-90%	1.52 (1.38, 1.69)	1.06 (1.03, 1.09)	1.21 (1.15, 1.27)	1.18 (1.11, 1.26)	1.14 (1.07, 1.21)	1.10 (1.03, 1.17)
90-100%	1.47 (1.35, 1.61)	1.10 (1.03, 1.09)	1.22 (1.17, 1.27)	1.16 (1.10, 1.22)	1.14 (1.09, 1.19)	1.14 (1.08, 1.20)
Population density (residents/mi^2)						
<1,000 (reference)	1	1	1	1	1	1
1,000-5000	0.84 (0.80, 0.88)	0.97 (0.95, 0.98)	0.88 (0.87, 0.90)	0.85 (0.82, 0.87)	0.86 (0.84, 0.88)	1.02 (0.99, 1.05)
+5,000	0.85 (0.80, 0.91)	0.97 (0.95, 0.99)	0.88 (0.85, 0.90)	0.84 (0.81, 0.88)	0.85 (0.82, 0.88)	1.00 (0.96, 1.04)
Population <5 years of age (%)	1.01 (1.01, 1.02)	1.00 (1.00, 1.00)	1.01 (1.01, 1.01)	1.01 (1.01, 1.02)	1.01 (1.01, 1.02)	1.00 (1.00, 1.01)
Population >64 years of age (%)	1.00 (0.99, 1.00)	1.00 (1.00, 1.00)	1.00 (1.00, 1.00)	1.00 (0.99, 1.00)	1.00 (1.00, 1.00)	1.01 (1.00, 1.01)
Residual Spatial Autocorrelation (Moran's I)	I = 0.04	I = 0.11	I = 0.07	I = 0.08	I = 0.21	I = -0.03

Table S1. Risk ratio (95% confidence interval) of six occupational indicators by tract-level percentage of racial and ethnic minority groups, population density, population less than 5 years of age, and population greater than 64 years of age (n = 2,708 MI census tracts). Results are shown in Figure 3 of the main text.

Quantile	OR (95% CI)	No. tracts at quantile
0.1	0.44 (0.35, 0.54)	49
0.15	0.46 (0.36, 0.55)	119
0.2	0.50 (0.41, 0.63)	154
0.25	0.57 (0.47, 0.69)	208
0.3	0.60 (0.51, 0.71)	224
0.35	0.75 (0.59, 0.89)	211
0.4	0.87 (0.78, 1.00)	194
0.45	0.93 (0.82, 1.02)	220
0.5 (reference)	1	193
0.55	1.05 (0.97, 1.19)	175
0.6	1.18 (1.05, 1.43)	150
0.65	1.26 (1.10, 1.48)	122
0.7	1.54 (1.29, 1.84)	113
0.75	1.81 (1.51, 2.23)	91
0.8	2.08 (1.72, 2.69)	113
0.85	2.72 (2.07, 3.49)	121
0.9	3.38 (2.72, 4.38)	136

Table S2. Odds ratio (95% confidence interval) of a census tract having high occupational and high environmental exposures (Cluster 3; n = 738/2,736) by simultaneous, quantile increases in the census tract-level percentage of racial and ethnic minority individuals, low-income individuals, and individuals without a high school diploma. Modeled using 100 Bootstraps of a gradient boosted regression tree grown 500 times, adjusting for the population density (residents/mi2), the percentage of individuals <5 years of age and >64 years of age, county, and the centroid of the census tract. Results are shown in Figure 4B of the main text. The number of census tracts with the average quantile of racial and ethnic minority individuals, low-income individuals, and individuals without a high school diploma is displayed in the final column.

Percent racial and ethnic minority	OR (95% CI)	Percent low-income	OR (95% CI)	Percent without HS diploma	OR (95% CI)
0 - 10% (reference)	1	0 - 10% (reference)	1	0 - 3% (reference)	1
10 - 20%	1.02 (0.97, 1.08)	10 - 20%	1.11 (1.03, 1.21)	3 - 6%	1.34 (1.17, 1.59)
20 - 30%	1.05 (0.97, 1.15)	20 - 30%	1.42 (1.26, 1.67)	6 - 9%	1.51 (1.29, 1.85)
30 - 40%	1.16 (1.04, 1.35)	30 - 40%	1.88 (1.61, 2.18)	9 - 12%	1.59 (1.31, 1.98)
40 - 50%	1.25 (1.10, 1.46)	40 - 50%	2.27 (1.95, 2.71)	12 - 15%	1.62 (1.32, 2.04)
50 - 60%	1.40 (1.16, 1.72)	50 - 60%	2.52 (2.14, 3.07)	15 - 16%	1.63 (1.31, 2.03)
60 - 70%	1.60 (1.26, 1.93)	60 - 71%	2.62 (2.14, 3.15)	--	--
70 - 80%	1.80 (1.45, 2.39)	--	--	--	--
80 - 90%	2.11 (1.67, 2.85)	--	--	--	--
90 - 97%	2.31 (1.78, 3.03)	--	--	--	--

Table S3. Odds ratio (95% confidence interval) of a census tract having high occupational and high environmental exposures (Cluster 3; n = 729/2,736) by increases in the census tract-level percentage of racial and ethnic minorities, low-income individuals, or individuals without a high school diploma. Modeled using 100 Bootstraps of a gradient boosted regression tree grown 500 times, adjusting for the population density (residents/mi2), the percentage of individuals <5 years of age and >64 years of age, county, and the centroid of the census tract. Results are shown in Figure 4C of the main text.

Characteristic	A<br>(n = 35)	B<br>(n = 106)	C<br>(n = 359)	D<br>(n = 229)	Overall<br>(n = 729)
Sociodemographic					
Racial and ethnic minority (% of population)	16.8% (9.1, 96.2)	63.8% (16.0, 97.5)	50.1% (21.8, 92.5)	73.1% (36.2, 92.7)	59.3% (23.8, 94.0)
Low-income (% of population)	17.3% (7.4, 52.4)	46.3% (24.4, 61.8)	53.5% (35.3, 66.6)	63.6% (48.0, 71.3)	55.3% (36.0, 67.4)
No HS diploma (% of population)	2.1% (1.2, 10.7)	6.3% (3.8, 9.9)	9.4% (6.0, 13.4)	12.0% (8.9, 17.7)	9.7% (5.9, 13.9)
Occupational					
Hazardous noise (% of working population)	6.3% (3.1, 13.7)	11.6% (8.1, 14.8)	14.4% (11.1, 17.6)	15.7% (12.0, 19.5)	14.1% (10.6, 17.7)
Physical proximity (in days)	82 (76, 92)	90 (81, 97)	90 (85, 97)	92 (86, 99)	91 (84, 97)
Chemical contaminant exposure (in days)	38 (32, 59)	55 (44, 65)	53 (52, 70)	64 (56, 73)	61 (52, 70)
Hazardous equipment use (in days)	19 (15, 31)	30 (23, 34)	35 (28, 40)	35 (29, 43)	34 (27, 39)
Hazardous conditions (in days)	16 (13, 22)	21 (17, 24)	24 (20, 27)	24 (21, 29)	23 (19, 27)
Disease/infection exposure (in days)	25 (21, 30)	25 (20, 29)	22 (18, 27)	21 (16, 26)	22 (18, 27)
Environmental					
PM2.5 (units)	9.3 (9.0, 9.5)	9.3 (8.0, 9.5)	9.3 (7.9, 9.5)	9.5 (8.2, 9.6)	9.3 (8.1, 9.5)
Diesel PM (units)	0.5 (0.4, 0.6)	0.6 (0.4, 0.7)	0.5 (0.3, 0.6)	0.6 (0.4, 0.7)	0.6 (0.4, 0.7)
Ozone (ppb)	44.3 (43.2, 45.2)	43.2 (42.7, 44.8)	43.5 (43.0, 44.6)	43.6 (43.0, 44.3)	43.5 (43.0, 44.5)
Air toxics cancer risk	28.2 (26.4, 28.7)	28.3 (25.5, 28.8)	28.2 (25.0, 29.2)	28.9 (25.3, 31.6)	28.4 (25.4, 29.4)
Respiratory hazard index	0.3 (0.3, 0.4)	0.3 (0.3, 0.4)	0.3 (0.3, 0.4)	0.4 (0.4, 0.4)	0.4 (0.3, 0.4)
Traffic volume	803 (455, 1478)	1228 (696, 2093)	1033 (600, 1768)	1255 (605, 2821)	1113 (604, 2104)
Transportation noise (% of population)	0% (0, 1.4)	0% (0, 2.1)	0% (0, 4.3)	0.8% (0, 8.6)	0.1% (0, 5.8)

Table S4. Sociodemographic, occupational indicator, and environmental indicator descriptive statistics (median, interquartile range) by HOLC grade.

Characteristic	A-C (n = 500)	D (n = 229)
Occupational		
Hazardous noise (% of working population)[a]	1 (Reference)	1.14 (1.05, 1.24)
Physical proximity (in days)[a]	1 (Reference)	1.02 (1.00, 1.04)
Chemical contaminant exposure (in days)[a]	1 (Reference)	1.09 (1.04, 1.13)
Hazardous equipment use (in days)[a]	1 (Reference)	1.10 (1.05, 1.16)
Hazardous conditions (in days)[a]	1 (Reference)	1.09 (1.04, 1.14)
Disease/infection exposure (in days)[a]	1 (Reference)	0.94 (0.89, 0.99)
Environmental		
$PM_{2.5}$ (units)[b]	0 (Reference)	0.02 (0.00, 0.03)
Diesel PM (units)[b]	0 (Reference)	0.02 (0.01, 0.03)
Ozone (ppb)[b]	0 (Reference)	-0.08 (-0.16, 0.01)
Air toxics cancer risk[c]	1 (Reference)	1.02 (1.01, 1.03)
Respiratory hazard index[b]	0 (Reference)	0.00 (0.00, 0.01)
Traffic volume[a]	1 (Reference)	1.07 (0.92, 1.24)
Transportation noise (% of population)[a]	1 (Reference)	1.58 (0.39, 6.4)

[a]Modeled using Poisson regression; reporting risk ratio.
[b]Modeled using linear regression; reporting change in units.
[c]Modeled using log-linear regression; reporting relative risk.

Table S5. Unadjusted effect estimates (95% CI) of six occupational and seven environmental hazards among historically redlined neighborhoods (D-grade) compared to non-redlined neighborhoods (grades A-C). All regressions use a mixed-effects conditional autoregressive model using queen-contiguity adjacency matrix with random intercept for city.

Quantile	OR (95% CI)	No. tracts at quantile
0.05	1.20 (0.62, 2.32)	0
0.1	1.10 (0.56, 1.72)	0
0.15	0.87 (0.49, 1.45)	1
0.2	0.79 (0.47, 1.32)	11
0.25	0.76 (0.48, 1.19)	28
0.3	0.83 (0.52, 1.33)	45
0.35	0.91 (0.59, 1.43)	70
0.4	1.23 (0.85, 1.84)	81
0.45	1.12 (0.89, 1.50)	74
0.5 (reference)	1	101
0.55	0.83 (0.64, 1.05)	81
0.6	0.87 (0.58, 1.33)	80
0.65	0.90 (0.54, 1.51)	68
0.7	0.94 (0.54, 1.67)	46
0.75	0.95 (0.55, 1.69)	34
0.8	1.35 (0.68, 2.69)	4
0.85	1.59 (0.81, 3.22)	2
0.9	2.47 (1.20, 5.36)	2
0.95	5.53 (2.65, 10.20)	0

Table S6. Odds ratio (95% confidence interval) of a census tract being historically redlined (HOLC Grade D; n = 224/722) by simultaneous, quantile increases in seven environmental indicators: air toxics cancer risk (units), diesel PM (units), ozone (units), PM2.5 (units), respiratory hazard index (units), traffic volume (units), and transportation noise indicator (units); and six occupational indicators: contaminant exposure (units), disease/infection exposure (units), hazardous workplace conditions (units), hazardous equipment use (units), prevalence of noise exposure (units), and close physical proximity (units). Modeled using 1,000 Bootstraps of a gradient boosted regression tree grown 500 times, adjusting for the percentage of low-income individuals and individuals without a high school diploma. Results are shown in Figure 5A of the main text. The number of census tracts with the average quantile of the thirteen environmental and occupational indicators is displayed in the final column.
